# Weakly imposed boundary conditions for shear-rate dependent non-Newtonian fluids: application to cardiovascular flows

**DOI:** 10.3934/mbe.2021193

**Published:** 2021-05-06

**Authors:** Soonpil Kang, Sharbel Nashar, Elizabeth R. Livingston, Arif Masud

**Affiliations:** Department of Civil and Environmental Engineering, and Department of Biomedical and Translational Sciences, University of Illinois at Urbana-Champaign, Urbana, IL 61801, USA

**Keywords:** weakly imposed Dirichlet boundary conditions, non-Newtonian shear-rate dependent fluids, Variational Multiscale method, interface stabilization, blood flows

## Abstract

This paper presents a stabilized formulation for the generalized Navier-Stokes equations for weak enforcement of essential boundary conditions. The non-Newtonian behavior of blood is modeled via shear-rate dependent constitutive equations. The boundary terms for weak enforcement of Dirichlet boundary conditions are derived via locally resolving the fine-scale variational equation facilitated by the Variational Multiscale (VMS) framework. The proposed method reproduces the consistency and stabilization terms that are present in the Nitsche type approaches. In addition, for the shear-rate fluids, two more boundary terms appear. One of these terms is the viscosity-derivative term and is a function of the shear-rate, while the other term is a zeroth-order term. These terms play an important role in attaining optimal convergence rates for the velocity and pressure fields in the norms considered. A most significant contribution is the form of the stabilization tensors that are also variationally derived. Employing edge functions the edge stabilization tensor is numerically evaluated, and it adaptively adjusts itself to the magnitude of the boundary residual. The resulting formulation is variationally consistent and the weakly imposed no-slip boundary condition leads to higher accuracy of the spatial gradients for coarse boundary-layer meshes when compared with the traditional strongly imposed boundary conditions. This feature of the present approach will be of significance in imposing interfacial continuity conditions across non-matching discretizations in blood-artery interaction problems. A set of test cases is presented to investigate the mathematical attributes of the method and a patient-specific case is presented to show its clinical relevance.

## 1. Introduction

Cardiovascular blood flow simulations of clinical relevance require non-Newtonian blood flow models and invariably involve complex patient-specific geometries with branching arterial trees. The quality of the computed solutions is affected by the precise description of the geometric models as well as the quality of the computational grids. The use of boundary layer meshes helps in accurately calculating flow quantities such as wall shear stress (WSS) and surface pressure at the arterial walls, and several mesh adaptation techniques have been proposed in the literature for local refinement along the boundaries [1–3]. This however comes at an increased computational cost due to the insertion of elements in the boundary layer region to achieve higher spatial resolution of the velocity and pressure gradients [1, 3]. In this context a numerical method that can yield higher spatio-temporal accuracy of the gradients in the fields and can facilitate the coupling of the primary fields across the blood-tissue interaction surfaces via weakly enforced continuity equation for the velocity field can open the doors for developing clinically relevant computational techniques.

The methods for weakly enforced no-slip conditions embed the Dirichlet boundary conditions in the variational formulation rather than prescribing the value of the Dirichlet data directly at the nodal points. This strategy allows the fluid particles to slightly slide at the wall and it helps in capturing the high gradients of the velocity field near the boundaries. These methodologies are also reported to help increase the accuracy of the boundary layer fields [4]. Similarly, methods for weakly imposed boundary conditions have also been proposed for wall-bounded turbulent flows [5] where they behave like wall function models and facilitate better performance as compared to the strongly imposed boundary conditions. In addition, the notion of weak imposition of boundary conditions has been effectively employed in advection-dominated diffusion problems [6], in porous flows [7], and in buoyancy-driven flows [8]. These studies suggest the possibility of being able to use coarse discretizations in arterial flow analysis with dominant presence of the confining bounding surfaces, thereby significantly reducing the mesh sizes due to the computational efficiency engendered by the weakly imposed boundary conditions.

Another advantage of the weakly imposed boundary conditions originates from the flexibility that these methods do not require nodally matched discretizations at the blood-artery interaction surfaces. The Dirichlet boundary is embedded in the variational equation via weakly imposed boundary conditions, thereby implicitly accounting for the continuity of the fields across the fluid-solid interface. Consequently, the computational meshes do not need to be nodally aligned, and this flexibility opens the door to develop methods for non-matching boundaries and interfaces [9–11] that are beneficial in complex geometries encountered in patient-specific models.

A literature review reveals that Nitsche-type methods have often been employed to variationally apply the no-slip boundary conditions [4, 7, 8, 11, 12]. The Nitsche method for constraining the primary unknown fields is comprised of the consistency terms and the penalty or stability term. A major technical issue has been the value of the stability parameter that largely remains unspecified by the theory. Since the condition number of the system is a function of the stability parameter, the optimal value of the parameter needs to be identified. This value needs to be small enough to preserve consistency of the method, while it needs to be sufficiently large for ensuring stability. If the parameter is too large, the stabilization term plays the role of penalizing the boundary conditions, and the formulation loses the variational consistency, thereby deteriorating the conditioning of the tangent matrix. If the value is too small, the formulation becomes unstable.

An important ingredient of the present paper is the use of shear-rate dependent non-Newtonian model for blood [13–15]. The flow of shear-thinning fluids generally gives rise to pseudo-plastic velocity profiles which is characterized by lower velocities due to a less convective flow but sharper and thinner boundary layers [15]. Therefore, the ability of the method to capture the boundary layer is of significant importance for the shear-thinning models than for the Newtonian models. The stabilized methods for this class of fluids were developed by the senior author, and the interested reader is referred to [15, 17–21].

This paper develops a stabilized formulation for the weak imposition of Dirichlet boundary conditions for non-Newtonian fluids. We derive the boundary terms by exploiting the edge-based functions that model fine scales in the Variational Multiscale (VMS) equations. Similar ideas have been applied to develop Discontinuous Galerkin (DG) methods for coupling multiple PDEs [22], in damage modeling for finite strain solid mechanics problems with weak and strong discontinuities across common interfaces [23], and in the immersed boundary conditions in fluid mechanics [10]. A unique contribution of the proposed method is the variationally derived consistency terms as well as the stabilization tensor that possesses a self-adjusting feature for optimal enforcement of the boundary conditions. This feature is highlighted with the help of numerical examples in Section 4.

An outline of the paper is as follows: Section 2 presents the governing equations and the constitutive model. Section 3 presents the derivation of the stabilized formulation. Section 4 presents numerical test cases to validate the proposed method and to investigate its mathematical and computational attributes. An application to patient-specific arterial geometry illustrates its clinical relevance. Conclusions are drawn in Section 5.

## 2. Governing equations and non-Newtonian fluid models

### 2.1. The strong form of governing equations

Let $\Omega \subset R^{n_{\text{sd }}}$ be an open bounded region with piecewise smooth boundary Γ. The number of spatial dimensions, $n_{\text{sd }}$, is equal to 2 or 3. The domain boundary assumes the usual split $\Gamma =\Gamma_{g}\cup \Gamma_{h}$ and $\Gamma_{g}\cap \Gamma_{h}=\emptyset$, where $\Gamma_{g}$ and $\Gamma_{h}$ are parts of the boundary with essential and natural boundary conditions, respectively. The governing equations and boundary conditions are given as:

(1) $$\rho \frac{\partial v}{\partial t}+\rho v\cdot \nabla v-\nabla \cdot \sigma_{v}\left(v\right)+\nabla p=\rho f\text{ in Ω}$$

(2) ∇⋅v=0   in Ω

(3) v=g   on Γg

(4) $$\sigma \cdot n=h{\text{ on Γ}}_{h}$$

where v and p are the fluid velocity and pressure, respectively. ρ is the density of the fluid, $\sigma_{v}$ is the deviatoric stress tensor, σ is the Cauchy stress tensor defined as $\sigma =-pI+\sigma_{v},I$ is the identity tensor, f is the body force per unit mass, g is the prescribed velocity on the boundary $\Gamma_{g},h$ is the prescribed traction on the boundary $\Gamma_{h}$, and n is the unit outward normal to the boundary of the domain Ω. Equations (1)–(4) represent balance of momentum, the continuity equation, the Dirichlet and Neumann boundary conditions, respectively. The shear-rate dependent deviatoric stress tensor is defined as

(5) $$\sigma_{v}=2\eta \left(\dot{\gamma }\right)\varepsilon \left(v\right)$$

where ε(v) is the rate-of-deformation tensor defined as $\varepsilon (v)=\frac{1}{2}\left(\nabla v+\nabla v^{T}\right),\dot{\gamma }$ is the shear-rate defined as $\dot{\gamma }=\sqrt{2\varepsilon (v):\varepsilon (v)}$, and $\eta (\dot{\gamma })$ is the effective viscosity which is a function of the shear-rate.

### 2.2. Non-Newtonian fluid models

In this work, we employ the power-law model and the Carreau-Yasuda model to represent the shear-thinning behavior of blood, which are the two commonly used models in computational hemodynamics [13–15,18,24]. The power-law model is:

(6) $$\eta \left(\dot{\gamma }\right)=\mu {\dot{\gamma }}^{n-1}$$

where μ represents the viscosity of Newtonian fluids if n=1. A drawback with this model is the singularity that appears at zero shear-rate, and various techniques have been proposed in the literature to establish a lower-bound on the effective viscosity.

In the Carreau-Yasuda model, the nonlinear function of the viscosity is given by

(7) $$\eta \left(\dot{\gamma }\right)=\mu_{\infty }+\left(\mu_{0}-\mu_{\infty }\right){\left[1+{\left(\lambda \dot{\gamma }\right)}^{a}\right]}^{\left(n-1\right)/a}$$

where $\mu_{0}$ and $\mu_{\infty }$ are asymptotic viscosities at zero and infinite shear-rate, respectively, and a,n and λ are empirically determined constitutive parameters. Coefficients a and n are non-dimensional parameters that control the shear-thinning or shear-thickening behavior of fluids in the non-Newtonian regime between the two asymptotic viscosities. The model reverts to the Newtonian fluid model by setting $\mu_{0}=\mu_{\infty }$.

### 2.3. The mixed weak formulation

Our objective is to develop the formulation that weakly imposes the Dirichlet boundary conditions for the velocity field in Eq (3). We start from the classical mixed formulation and derive the expression for the Lagrange multiplier field at the Dirichlet boundary $\Gamma_{g}$. Let w and q denote the weighting functions for the velocity and the pressure fields, and ψ denotes the weighting function for the boundary condition. The appropriate spaces of trial solutions and weighting functions are specified as follows:

(8) $$𝒮=\left\{v|v\in {\left[H^{1}\left(\Omega \right)\right]}^{n_{\text{sd}}},v=g{\text{ on Γ}}_{g}\right\}$$

(9) $$𝒱=\left\{w|w\in {\left[H^{1}\left(\Omega \right)\right]}^{n_{\text{sd}}},w=0{\text{ on Γ}}_{g}\right\}$$

(10) $$𝒫=\left\{p|p\in L_{2}\left(\Omega \right)\right\}$$

(11) $$𝒬=\left\{\lambda |\lambda \in {\left[H^{-1/2}\left(\Gamma_{g}\right)\right]}^{n_{\text{sd}}}\right\}$$

The mixed weak form associated with Eqs (1)–(4) is: Find {v,p}∈𝒮×𝒫,λ∈𝒬 such that for all {w,q}∈𝒱×𝒫,ψ∈𝒬 :

(12) $${\left(w,\rho \frac{\partial v}{\partial t}\right)}_{\Omega }+{\left(w,\rho v\cdot \nabla v\right)}_{\Omega }+{\left(\nabla w,2\eta \left(\dot{\gamma }\right)\varepsilon \left(v\right)\right)}_{\Omega }-{\left(\nabla \cdot w,p\right)}_{\Omega }+{\left(q,\nabla \cdot v\right)}_{\Omega \;}+{\left(w,\lambda \right)}_{\Gamma_{g}}={\left(w,h\right)}_{\Gamma_{h}}+{\left(w,\rho f\right)}_{\Omega }$$

(13) $${\left(\psi ,v-g\right)}_{\Gamma_{g}}=0$$

where $(\cdot ,\cdot {)}_{\Omega }=\int_{\Omega }\;(\cdot )\;d\Omega$ is the $L_{2}(\Omega )$ inner product. In this work shear-rate dependent deviatoric stress is considered. The mixed formulation in Eqs (12) and (13) consistently enforces the boundary condition Eq (3) through the Lagrange multiplier λ which acts as the numerical flux or the traction at $\Gamma_{g}$. The downside of the mixed formulation is the presence of additional unknown fields and the instability associated with the inf-sup conditions. To overcome these issues while holding the virtue of variational consistency, we derive the expression for the Lagrange multiplier λ that will eliminate the auxiliary unknown field from the formulation.

## 3. The stabilized formulation for weakly imposed boundary conditions

**Figure 1.**
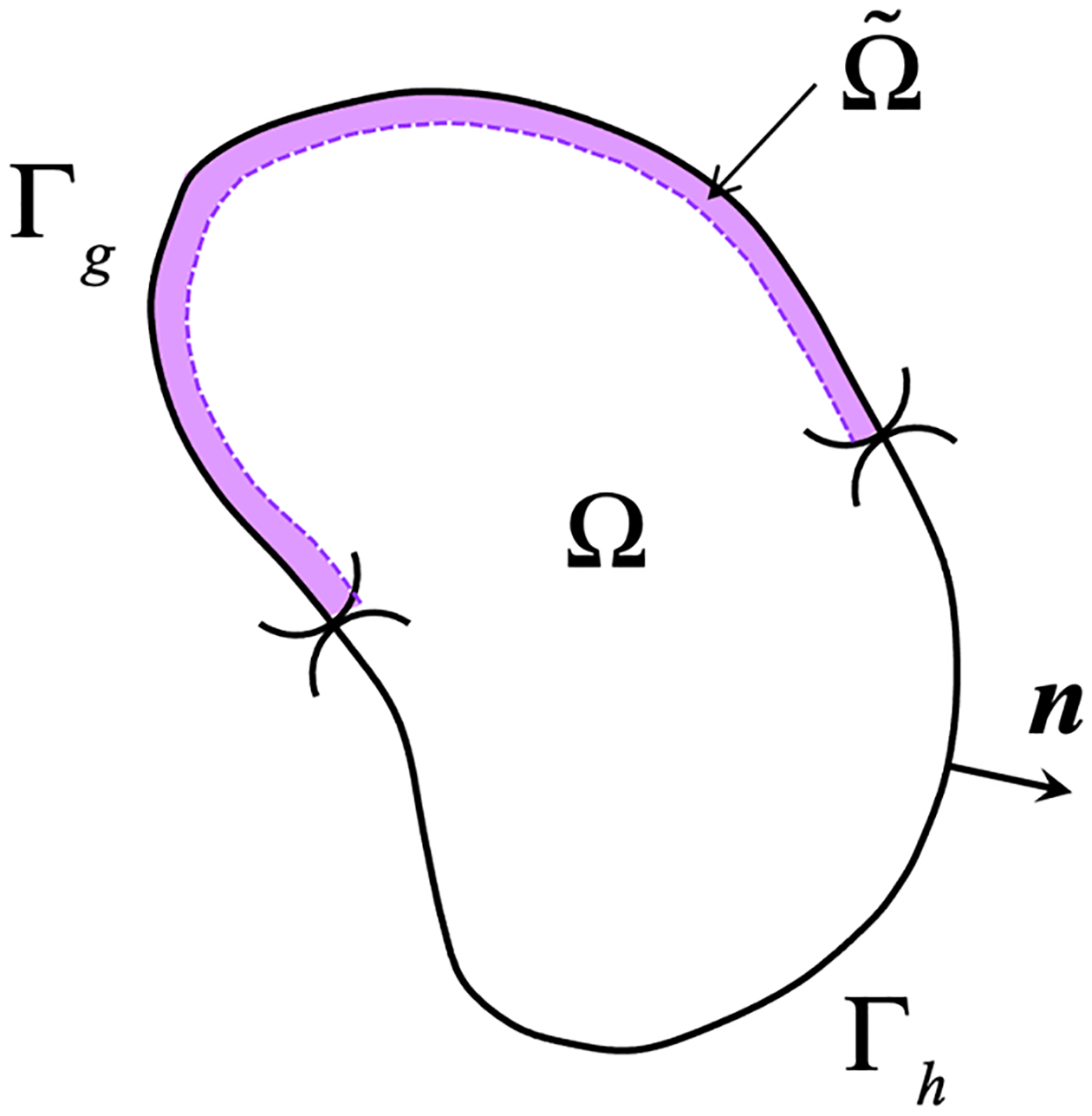
Narrow band along the Dirichlet boundary where the fine-scale field is active.

This section presents the derivation of the stabilized formulation for the weakly imposed Dirichlet boundary conditions. We apply the VMS framework to the narrow band $\tilde{\Omega }\subset \Omega$ along the Dirichlet boundary $\Gamma_{g}$ shown in Figure 1. Overall procedure underlying the derivation comprises three steps, (i) split the problem into coarse- and fine-scale sub-problems, (ii) solve the fine-scale problem along the boundary $\Gamma_{g}$, and (iii) embed the fine-scale models into the coarse-scale formulation.

### 3.1. Multiscale decomposition

We discretize the domain Ω into disjoint elements $\Omega^{e}$ with element boundaries $\Gamma^{e}$, such that $\Omega ={⋃}_{e=1}^{n_{\text{el}}}\;\Omega^{e}$ where $n_{\text{el}}$ is the total number of elements in the mesh. We apply a multiscale overlapping decomposition to the velocity field and the weighting functions only in the narrow band $\tilde{\Omega }$ along the Dirichlet boundary.

(14) $$v=\overset{ˆ}{v}+\overset{˜}{v}$$

(15) $$w=\overset{ˆ}{w}+\overset{˜}{w}$$

where $\overset{ˆ}{v}$ and $\tilde{v}$ are the coarse and fine scale velocity fields, respectively. $\overset{ˆ}{w}$ and $\tilde{w}$ are the corresponding weighting functions for the coarse and fine scales, respectively. The coarse scale is associated with finite element spaces and the fine scale is represented by piecewise polynomials of sufficiently high order. Substituting Eqs (14) and (15) into the mixed weak form in Eqs (12) and (13) and using the linearity of the bilinear forms, we obtain two variational forms:

#### Coarse-scale sub-problem


(16) $${\left(\overset{ˆ}{w},\rho \frac{\partial \left(\overset{ˆ}{v}+\overset{˜}{v}\right)}{\partial t}\right)}_{\Omega }+{\left(\overset{ˆ}{w},\rho \left(\overset{ˆ}{v}+\overset{˜}{v}\right)\cdot \nabla \left(\overset{ˆ}{v}+\overset{˜}{v}\right)\right)}_{\Omega }+{\left(\nabla \overset{ˆ}{w},2\eta \left(\dot{\gamma }\right)\varepsilon \left(\overset{ˆ}{v}+\overset{˜}{v}\right)\right)}_{\Omega }-{\left(\nabla \cdot \overset{ˆ}{w},p\right)}_{\Omega }+{\left(q,\nabla \cdot \left(\overset{ˆ}{v}+\overset{˜}{v}\right)\right)}_{\Omega }+{\left(\overset{ˆ}{w},\lambda \right)}_{\Gamma_{g}}={\left(\overset{ˆ}{w},h\right)}_{\Gamma_{h}}+{\left(\overset{ˆ}{w},\rho f\right)}_{\Omega }$$

(17) $${\left(\psi ,\overset{ˆ}{v}+\overset{˜}{v}-g\right)}_{\Gamma_{g}}=0$$

#### Fine-scale sub-problem


(18) $${\left(\overset{˜}{w},\rho \frac{\partial \left(\overset{ˆ}{v}+\overset{˜}{v}\right)}{\partial t}\right)}_{\Omega }+{\left(\overset{˜}{w},\rho \left(\overset{ˆ}{v}+\overset{˜}{v}\right)\cdot \nabla \left(\overset{ˆ}{v}+\overset{˜}{v}\right)\right)}_{\Omega }+{\left(\nabla \overset{˜}{w},2\eta \left(\dot{\gamma }\right)\varepsilon \left(\hat{v}+\overset{˜}{v}\right)\right)}_{\Omega }\;-{\left(\nabla \cdot \overset{˜}{w},p\right)}_{\Omega }+{\left(\overset{˜}{w},\lambda \right)}_{\Gamma_{g}}={\left(\overset{˜}{w},h\right)}_{\Gamma_{h}}+{\left(\overset{˜}{w},\rho f\right)}_{\Omega }$$

The coarse-scale sub-problem governs the computable scales along $\Gamma_{g}$, while the fine-scale sub-problem governs the residual-based error part along $\Gamma_{g}$. Because we apply the scale decomposition only at $\tilde{\Omega }$, the fine-scale problem Eq (18) is localized around the Dirichlet boundary $\Gamma_{g}$. Accordingly, the fine scales $\tilde{v}$ are assumed to be non-zero only within elements at the boundary $\Gamma_{g}$ and they vanish in the interior of the domain Ω.

### 3.2. Derivation of the fine-scale models

**Figure 2.**
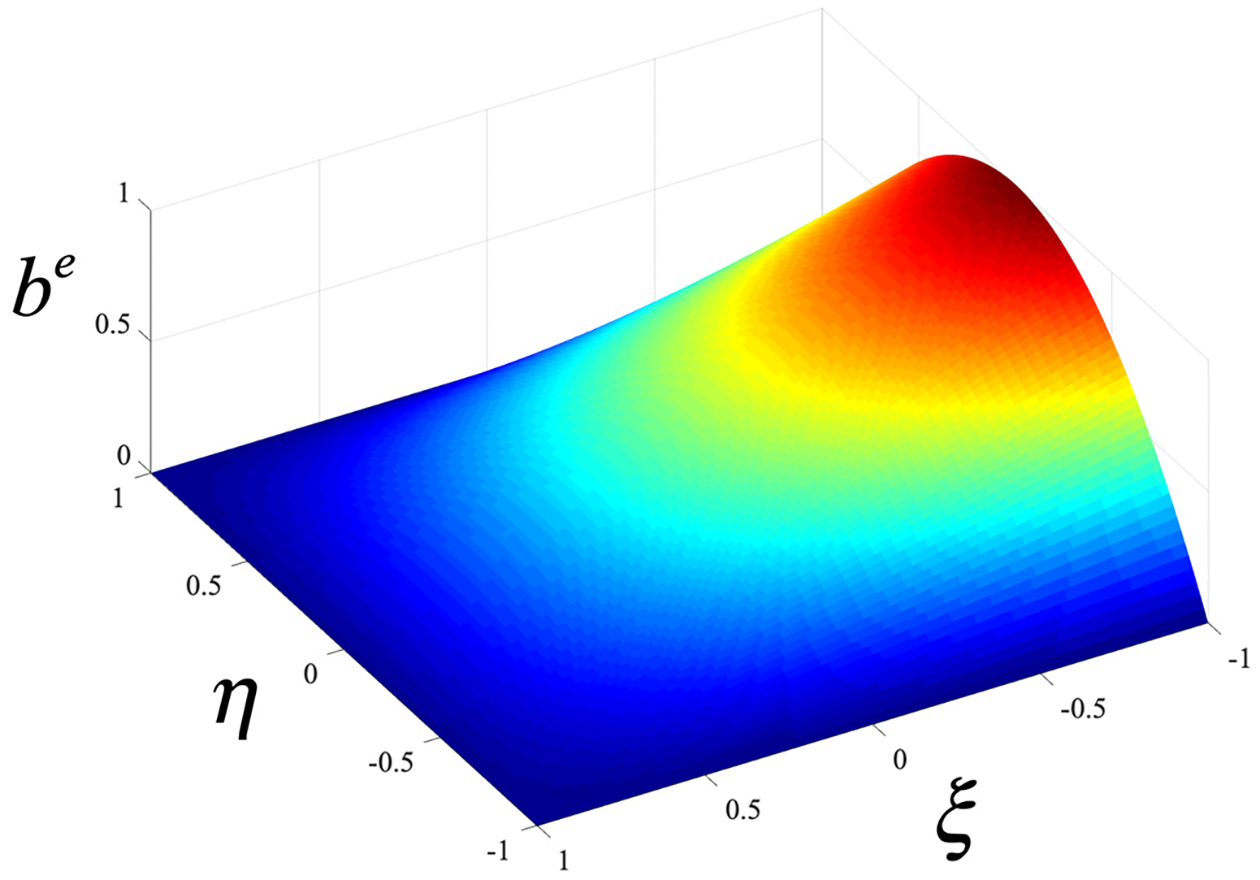
Edge function $b^{e}$.

We assume that the fine-scale field is operational over the first layer of elements that are adjacent to the boundary $\Gamma_{g}$. Consequently, it is nonzero on $\tilde{\Omega }$, attaining maximum value at $\Gamma_{g}$ and becoming zero at distance ε=h perpendicular to $\Gamma_{g}$, where h is the width of the element normal to the boundary. This modeling assumption allows the continuity of the overlapping coarse and fine fields. It also yields a simpler method for numerical integration and therefore for the approximation of the conditions at the edges. We approximate the fine-scale fields using edge functions $b^{e}(\xi )$ defined in the natural coordinate system ξ=(ξ,η,ζ). They are non-zero at the segment of Dirichlet boundaries $\Gamma_{g}^{e}$ and zero at the other element boundaries. An example of edge function is shown in Figure 2 and Table 1 lists the edge functions for 2D and 3D elements. The fine scales are represented as follows:

(19) $$\overset{˜}{v}=b^{e}\alpha$$

(20) $$\overset{˜}{w}=b^{e}\beta$$

where α and β is the coefficient for the trial solutions and weighting functions of the fine scale velocities, respectively. We make a simplifying assumption that $\tilde{v}$ is quasi-static so that the effect of its time-derivative is negligible, i.e., $\partial \tilde{v}/\partial t\approx 0$.

We linearize the fine-scale problem with respect to $\tilde{v}$ by applying the variational derivative, $D_{v}[G]\cdot \Delta v=\frac{\partial }{\partial \varepsilon }[G(v+\varepsilon \Delta v){]}_{\varepsilon =0}$. We gather the coarse-scale terms on the right-hand side and apply integration-by-parts and the divergence theorem to them. The resulting linearized problem for the incremental fine scales $\Delta \tilde{v}$ is

(21) $${\left(\overset{˜}{w},\rho \left(\Delta \overset{˜}{v}\right)\cdot \nabla v\right)}_{\tilde{\Omega }}+{\left(\overset{˜}{w},\rho v\cdot \nabla \left(\Delta \overset{˜}{v}\right)\right)}_{\tilde{\Omega }}+{\left(\nabla \overset{˜}{w},2\eta \left(\dot{\gamma }\right)\varepsilon \left(\Delta \overset{˜}{v}\right)\right)}_{\tilde{\Omega }}+{\left(\nabla \overset{˜}{w},2\left\{D_{\tilde{v}}\left[\eta \left(\dot{\gamma }\right)\right]\cdot \Delta \overset{˜}{v}\right\}\varepsilon \left(v\right)\right)}_{\tilde{\Omega }}=-{\left(\overset{˜}{w},r\right)}_{\tilde{\Omega }}-{\left(\overset{˜}{w},\sigma \cdot n+\lambda \right)}_{\Gamma_{g}}-{\left(\overset{˜}{w},\sigma \cdot n-h\right)}_{\Gamma_{h}}$$

where r is the residual of the Euler Lagrange equations of the coarse scale formulation, and is given as

(22) $$r=\rho \frac{\partial v}{\partial t}+\rho v\cdot \nabla v-\nabla \cdot \sigma_{v}\left(v\right)+\nabla p-\rho f$$

The variation of the viscosity field is expressed as

(23) $$D_{\overset{˜}{v}}\left[\eta \left(\dot{\gamma }\right)\right]\cdot \Delta \overset{˜}{v}=\eta^{\prime }\left(\dot{\gamma }\right)D_{\overset{˜}{v}}\left[\dot{\gamma }\right]\cdot \Delta \overset{˜}{v}\;=\eta^{\prime }\left(\dot{\gamma }\right)\frac{1}{2}{\left(2\varepsilon \left(v\right):\varepsilon \left(v\right)\right)}^{-1/2}D_{\overset{˜}{v}}\left[2\varepsilon \left(v\right):\varepsilon \left(v\right)\right]\cdot \Delta \overset{˜}{v}\;=2\eta^{\prime }\left(\dot{\gamma }\right){\dot{\gamma }}^{-1}\left(\varepsilon \left(v\right):\varepsilon \left(\Delta \overset{˜}{v}\right)\right)$$

By substituting expressions Eqs (19), (20) and (23) into Eq (21), the fine-scale problem can be segregated into local problems over the elements $\Omega^{e}$ that lie along the Dirichlet boundaries $\Gamma_{g}$.


(24) $${\left(b^{e}\beta ,\rho \left(b^{e}\Delta \alpha \right)\cdot \nabla v\right)}_{\Omega^{e}}+{\left(b^{e}\beta ,\rho v\cdot \nabla \left(b^{e}\Delta \alpha \right)\right)}_{\Omega^{e}}+{\left(\nabla b^{e}\beta ,2\eta \left(\dot{\gamma }\right)\varepsilon \left(b^{e}\Delta \alpha \right)\right)}_{\Omega^{e}}+{\left(\nabla b^{e}\beta ,4\eta^{\prime }\left(\dot{\gamma }\right){\dot{\gamma }}^{-1}\left(\varepsilon \left(v\right):\varepsilon \left(b^{e}\Delta \alpha \right)\right)\varepsilon \left(v\right)\right)}_{\Omega^{e}}=-{\left(b^{e}\beta ,r\right)}_{\Omega^{e}}-{\left(b^{e}\beta ,\sigma \cdot n+\lambda \right)}_{\Gamma_{g}^{e}}-{\left(b^{e}\beta ,\sigma \cdot n-h\right)}_{\Gamma_{h}^{e}}$$

We solve the linear problem for the fine-scale coefficients Δα and get the closed form expression for the fine-scale velocity field.

(25) $$\Delta \overset{˜}{v}=-b^{e}\overset{˜}{\tau }\left[\int_{\Omega^{e}}b^{e}r\;d\Omega +\int_{\Gamma_{g}^{e}}b^{e}\left(\sigma \cdot n+\lambda \right)\;d\Gamma +\int_{\Gamma_{h}^{e}}b^{e}\left(\sigma \cdot n-h\right)\;d\Gamma \right]$$

where

(26) $$\overset{˜}{\tau }=\int_{\Omega^{e}}\rho {\left(b^{e}\right)}^{2}\nabla v^{T}\;d\Omega +\int_{\Omega^{e}}\rho b^{e}v\cdot \nabla b^{e}\;d\Omega I+\int_{\Omega^{e}}\eta \left(\dot{\gamma }\right)\left(\nabla b^{e}⊗\nabla b^{e}\right)\;d\Omega +\int_{\Omega^{e}}\eta \left(\dot{\gamma }\right){\left|\nabla b^{e}\right|}^{2}\;d\Omega I+\int_{\Omega^{e}}4\eta^{\prime }\left(\dot{\gamma }\right){\dot{\gamma }}^{-1}\left(\varepsilon \left(v\right)\cdot \nabla b^{e}\right)⊗\left(\nabla b^{e}\cdot \varepsilon \left(v\right)\right)\;d\Omega$$

The last boundary term in Eq (25) vanishes because the edge function $b^{e}$ is zero at the element edges on the traction boundary $\Gamma_{h}^{e}$. To further simplify the fine-scale expression Eq (25), we employ three modeling assumptions [22, 25]. (i) The edge bubble function $b^{e}$ is orthogonal to the coarse-scale residual r, so the domain-interior term in Eq (25) is neglected, i.e., $\int_{\Omega^{e}}\;b^{e}r\;d\Omega \approx 0$. Consequently, the fine-scale problem is driven by the boundary residual at the Dirichlet boundary $\Gamma_{g}^{e}$. (ii) The boundary residual is taken out of the integral by applying the mean-value theorem, i.e., $\int_{\Gamma_{g}^{e}}\;b^{e}(\sigma \cdot n+\lambda )\;d\Gamma \approx (\sigma \cdot n+\lambda )\int_{\Gamma_{g}^{e}}\;b^{e}\;d\Gamma$. (iii) The edge function is averaged along the boundary, i.e., $b^{e}\approx {\left[\text{meas}\left(\Gamma_{g}^{e}\right)\right]}^{-1}\int_{\Gamma_{g}^{e}}\;b^{e}\;d\Gamma$ where meas $\left(\Gamma_{g}^{e}\right)$ is the length or the area of the element edge on $\Gamma_{g}^{e}$. With these modeling assumptions, we arrive at the fine-scale model at the boundary $\Gamma_{g}^{e}$ that is driven by the boundary residual of the corresponding coarse scales.

(27) $$\Delta \overset{˜}{v}=-\tau_{g}^{-1}\left(\sigma \cdot n+\lambda \right)$$

where the stability tensor is defined as

(28) $$\tau_{g}=\left[\text{meas}\left(\Gamma_{g}^{e}\right)\right]{\left(\int_{\Gamma_{g}^{e}}b^{e}\;d\Gamma \right)}^{-2}\overset{˜}{\tau }$$

where $\tilde{\tau }$ is given by Eq (26).

**Remark**: *The last term in* Eq (26) *involves spatial gradient of nonlinear viscosity. This term would drop out for element-wise constant viscosity over a boundary element* [19]. *To keep the formulation general, we assume the viscosity to vary spatially over the element, and consequently we keep this term in the definition of the stability tensor*.

### 3.3. Variational embedding in the coarse-scale formulation

In Section 3.2, we derived the fine-scale model for the incremental velocity field along the Dirichlet boundaries $\Gamma_{g}$. The fine-scale velocity in Eq (27) is expressed in terms of the Lagrange multiplier λ which is still an unknown field. To derive a closed-form expression for λ, we substitute Eq (27) into Eq (17).

(29) $${\left(\psi ,v-g-\tau_{g}^{-1}\left(\sigma \cdot n+\lambda \right)\right)}_{\Gamma_{g}^{e}}=0$$

Assuming a piecewise constant projection of λ along $\Gamma_{g}^{e}$, we solve Eq (29) locally to obtain the expression for the Lagrange multiplier λ.


(30) $$\lambda =-\sigma \cdot n+\tau_{g}\left(v-g\right)$$

Notice that the Lagrange multiplier comprises the Cauchy traction and a penalty force to enforce the boundary condition. Substitution of Eq (30) in Eq (27) leads to an explicit form of the fine-scale model.


(31) $$\Delta \overset{˜}{v}=-\left(v-g\right)$$

The fine scale in the current framework can be viewed as the residual or the error of the Dirichlet boundary condition Eq (3) at the boundary $\Gamma_{g}$.

To embed the fine-scale model, we linearize the coarse-scale formulation Eq (16) with respect to the fine-scale velocity field $\tilde{v}$.


(32) $${\left(\overset{ˆ}{w},\rho \frac{\partial \overset{ˆ}{v}}{\partial t}\right)}_{\Omega }+{\left(\overset{ˆ}{w},\rho v\cdot \nabla v\right)}_{\Omega }+{\left(\nabla \overset{ˆ}{w},2\eta \left(\dot{\gamma }\right)\varepsilon \left(v\right)\right)}_{\Omega }-{\left(\nabla \cdot \overset{ˆ}{w},p\right)}_{\Omega }+{\left(q,\nabla \cdot v\right)}_{\Omega }+{\left(\overset{ˆ}{w},\lambda \right)}_{\Gamma_{g}}+{\left(\overset{ˆ}{w},\rho \left(\Delta \overset{˜}{v}\right)\cdot \nabla v\right)}_{\tilde{\Omega }}+{\left(\overset{ˆ}{w},\rho v\cdot \nabla \left(\Delta \overset{˜}{v}\right)\right)}_{\tilde{\Omega }}+{\left(\nabla \overset{ˆ}{w},2\eta \left(\dot{\gamma }\right)\varepsilon \left(\Delta \overset{˜}{v}\right)\right)}_{\tilde{\Omega }}+{\left(\nabla \overset{ˆ}{w},4\eta^{\prime }\left(\dot{\gamma }\right){\dot{\gamma }}^{-1}\left(\varepsilon \left(v\right):\varepsilon \left(\Delta \overset{˜}{v}\right)\right)\varepsilon \left(v\right)\right)}_{\tilde{\Omega }}+{\left(q,\nabla \cdot \left(\Delta \overset{˜}{v}\right)\right)}_{\tilde{\Omega }}={\left(\overset{ˆ}{w},h\right)}_{\Gamma_{h}}+{\left(\overset{ˆ}{w},\rho f\right)}_{\Omega }$$

To convert the fine-scale terms that are integrated over the narrow band $\tilde{\Omega }$ into the boundary terms, we apply integration-by-parts. We keep only the boundary contributions and neglect the interior terms following the assumption that fine-scales are only active at the boundary $\Gamma_{g}$. Substituting Eqs (30) and (31) in the linearized form Eq (32) yields the stabilized boundary formulation. The technical details of the steps for conversion from the domain-interior terms in $\tilde{\Omega }$ to the boundary terms on $\Gamma_{g}$ are provided in Appendix A. Furthermore, we add the residual-based interior stabilization $(\chi ,\tau r{)}_{\Omega }$ for flows of the shear-rate dependent fluids developed in [18, 19]. Hereon, we drop the superposed hats from the coarse-scale velocity field and its weighting function.

(33) $${\left(w,\rho \frac{\partial v}{\partial t}\right)}_{\Omega }+{\left(w,\rho v\cdot \nabla v\right)}_{\Omega }+{\left(\nabla w,2\eta \left(\dot{\gamma }\right)\varepsilon \left(v\right)\right)}_{\Omega }-{\left(\nabla \cdot w,p\right)}_{\Omega }+{\left(q,\nabla \cdot v\right)}_{\Omega }+{\left(\chi ,\tau r\right)}_{\Omega }-{\left(w,\sigma \cdot n\right)}_{\Gamma_{g}}-{\left(\rho \left(v\cdot n\right)w,v-g\right)}_{\Gamma_{g}}-{\left(2\eta \left(\dot{\gamma }\right)\varepsilon \left(v\right)\cdot n+qn,v-g\right)}_{\Gamma_{g}}+{\left(4\eta^{\prime }\left(\dot{\gamma }\right){\dot{\gamma }}^{-1}\left(\nabla w:\varepsilon \left(v\right)\right)n\cdot \varepsilon \left(v\right),v-g\right)}_{\Gamma_{g}}+{\left(w,\tau_{g}\left(v-g\right)\right)}_{\Gamma_{g}}={\left(w,h\right)}_{\Gamma_{h}}+{\left(w,\rho f\right)}_{\Omega }$$

Where

(34) $$\chi =\rho \left(-\nabla v\cdot w+\left(\nabla \cdot v\right)w+v\cdot \nabla w\right)+\eta \left(\dot{\gamma }\right)(\nabla \left(\nabla \cdot w\right)+\Delta w\;+\nabla \eta \left(\dot{\gamma }\right)\cdot \left(\left(\nabla \cdot w\right)I+\nabla w\right)+\nabla q$$

(35) $$\tau =b\int_{\Omega^{e}}b\;d\Omega {\left[\begin{array}{l} \int_{\Omega^{e}}\rho b^{2}\nabla v^{T}\;d\Omega +\int_{\Omega^{e}}\rho bv\cdot \nabla b\;d\Omega I\\ +\int_{\Omega^{e}}\eta \left(\dot{\gamma }\right)\left(\nabla b⊗\nabla b\right)\;d\Omega +\int_{\Omega^{e}}\eta \left(\dot{\gamma }\right)|\nabla b{|}^{2}\;d\Omega I \end{array}\right]}^{-1}$$

Note that the stability tensor τ is computed using the regular bubble function b(ξ), while the boundary stability tensor $\tau_{g}$ is computed using the edge function $b^{e}(\xi )$.

In Eq (33), the stabilized formulation is augmented by the weakly imposed boundary condition. The boundary terms integrated over $\Gamma_{g}$ weakly enforce the Dirichlet boundary condition. The first three boundary terms ensure the consistency of the formulation, which is required for optimal convergence. The fourth boundary term emanates from the linearization of the shear stress term of the fine-scale problem, which disappears for the Newtonian fluid case. The last boundary term stabilizes the boundary formulation, where the stability tensor is self-calculated without user-parameter.

**Remark**: *The derived formulation* Eq (33) *can be used with any shear-rate dependent fluid model. In this work, we have employed the power-law and the Carreau-Yasuda models. The formulation also accommodates the degenerate case of Newtonian fluids where the viscosity becomes constant and the viscosity-gradient term drops out*.

**Remark**: *An important point to note is that all the boundary terms are variationally derived and therefore the resulting formulation* Eq (33) *is variationally consistent*.

**Remark**: *The fourth boundary term in the formulation* Eq (33) *involving the gradient of viscosity emanates from the linearization of the fine-scale problem. The effects of this term on the convergence of the formulation are discussed in* Section 4.1.2. *In the numerical experiment of shear-thinning flow in a curved tube discussed in* Section 4.3.2, *simulations without this term diverge unless the time-step size is substantially reduced. It shows that this term helps in preserving unconditional stability of the implicit time integration methods*.

**Remark**: *The fourth boundary term in the formulation* *Eq* (33) *involves the inverse of the shear-rate, which can cause singularity at* $\dot{\gamma }\approx 0$. *The boundedness of this term is shown in* Appendix B. *In our numerical implementation, this boundary term is deactivated if* $\dot{\gamma }<\varepsilon$ *where* $\varepsilon =10^{-16}$.

## 4. Numerical results

We have implemented the stabilized boundary formulation Eq (33) and its consistent tangent tensors using linear quadrilateral elements for 2D problems and quadratic tetrahedral elements for 3D problems. The generalized-α method is used for time integration. It is an implicit, second-order accurate scheme with a free parameter $0\leq \rho_{\infty }\leq 1$ that controls the damping of high frequency modes. In this work, we have used $\rho_{\infty }=0.5$ for all the transient test cases. The nonlinear problems are solved using the Newton-Raphson method with the consistent tangent. We solve the linear system of equations using the direct solver for convergence tests and 2D cavity flow, and the GMRES solver with additive Schwarz preconditioner for the 3D arterial flow test cases.

### 4.1. Rate of convergence study

#### 4.1.1. Domain with no-penetration boundaries

This test case [18] investigates the convergence-rates for the strongly and weakly imposed boundary conditions. The domain is a 3D block, -0.5≤x,y,z≤0.5, and it is discretized using evenly spaced quadratic tetrahedral elements. The number of elements per edge is N=2,4,8,16,32. The exact solution for the velocity and pressure fields is given as follows

(36) $$v=\left[\begin{array}{c} -2z\text{cos}\left(\pi x\right)\text{cos}\left(\pi y\right)e^{\pi (-z^{2}+0.25),}\\ -2z\text{cos}\left(\pi x\right)\text{cos}\left(\pi y\right)e^{\pi (-z^{2}+0.25),}\\ \text{sin}\left(\pi \left(x+y\right)\right)\left(e^{\pi \left(-z^{2}+0.25\right)}-1\right) \end{array}\right]$$

(37) p=sin(2πx)sin(2πy)sin(2πz)

Note that the velocity field is divergence-free. The corresponding body force for the exact velocity and pressure fields is given in Appendix A in [18].

The density of the fluid is $=1.0\text{ kg}\cdot {\text{m}}^{-3}$. The material parameters for Carreau-Yasuda models are set as $\mu_{\infty }=0.00345\text{ Pa}\cdot \text{s},\mu_{0}=0.056\text{ Pa}\cdot \text{s},\lambda =1.902,n=0.22$ and a=1.25. Based on the maximum velocity and the viscosity at the infinite shear-rate $\mu_{\infty }$, the Reynolds number is Re=435. The flow is driven by the body force given in [18]. The values of the exact velocities are applied as nodal coefficients on all boundaries for the case of strongly imposed boundary conditions, while they are enforced via the formulation in Eq (33) for the weakly imposed boundary conditions.

**Figure 3.**
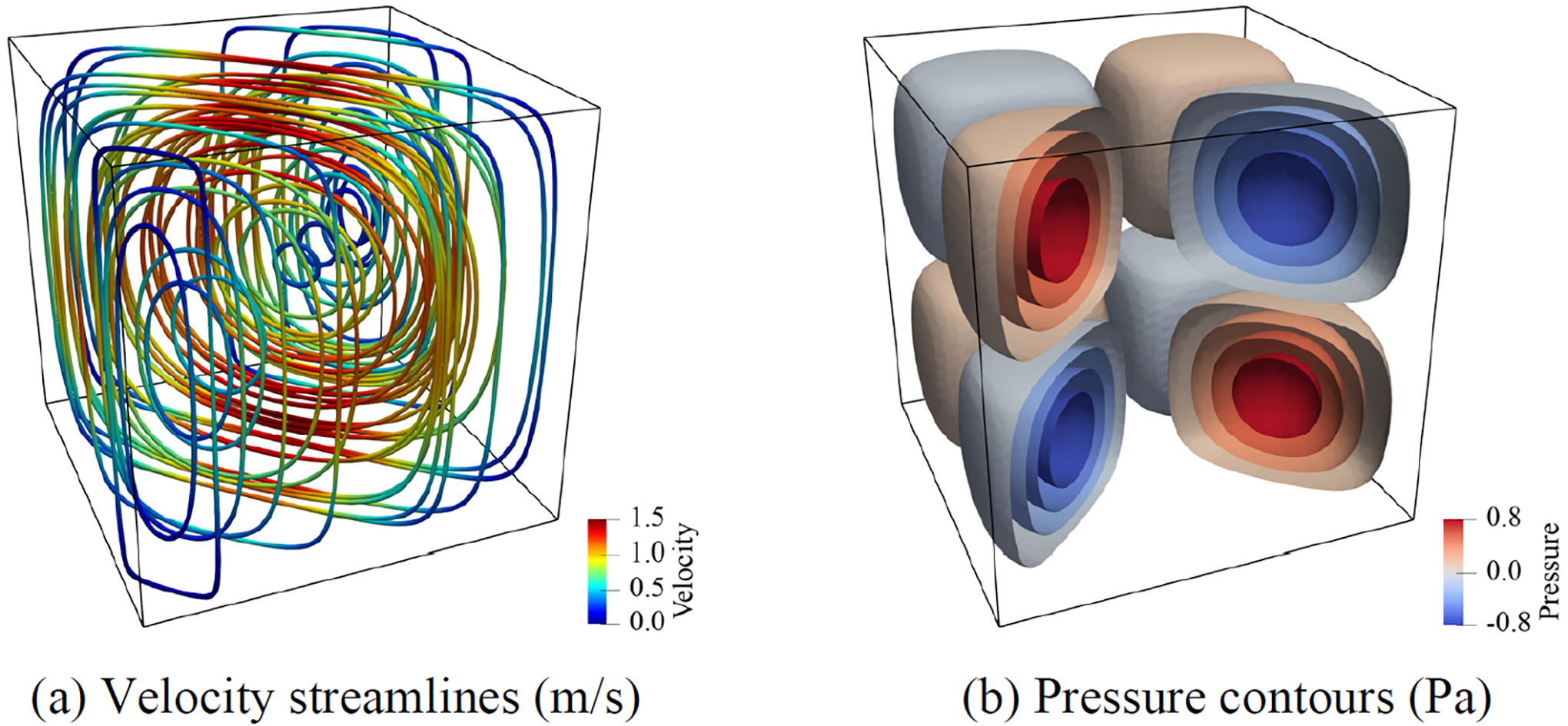
Computed streamlines and pressure contours in the domain -0.5≤x,y,z≤0.5.

**Figure 4.**
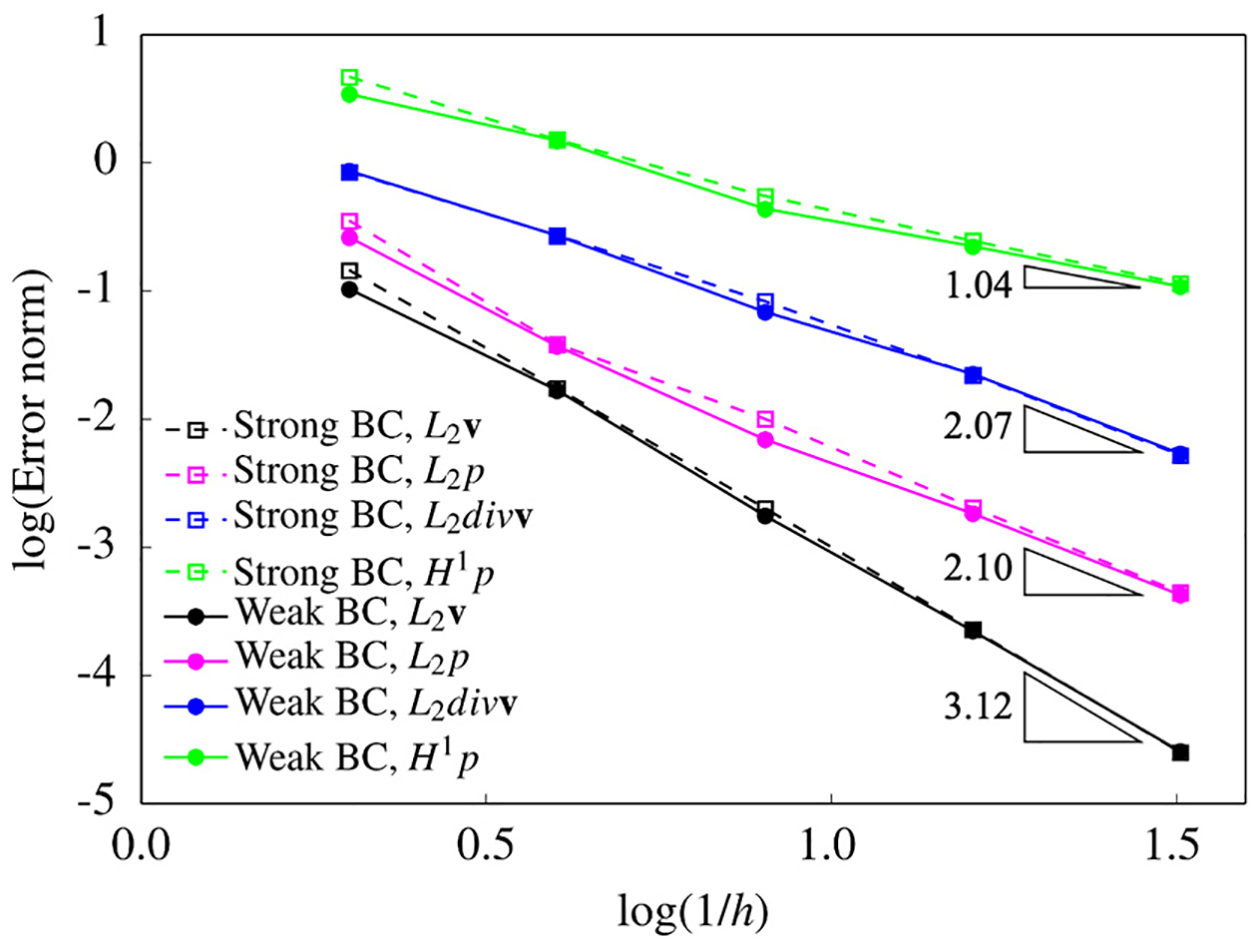
Rate of convergence study for the quadratic tetrahedral elements (h is the side length of the element).

Figure 3 presents the velocity and pressure fields computed on the finest mesh with the weakly imposed boundary conditions. Figure 4 shows the convergence rates for the $L_{2}$-norm and the $H^{1}$ seminorm of error that shows optimal convergence rates for the velocity field and its divergence. Both strong and weak boundary conditions show equivalent convergence properties for the finer meshes, while the weak boundary condition results in smaller error for the coarse mesh. This shows that the weakly imposed boundary conditions help improve the accuracy of calculations on cruder spatial discretizations.

#### 4.1.2. Domain with penetration boundaries

**Figure 5.**
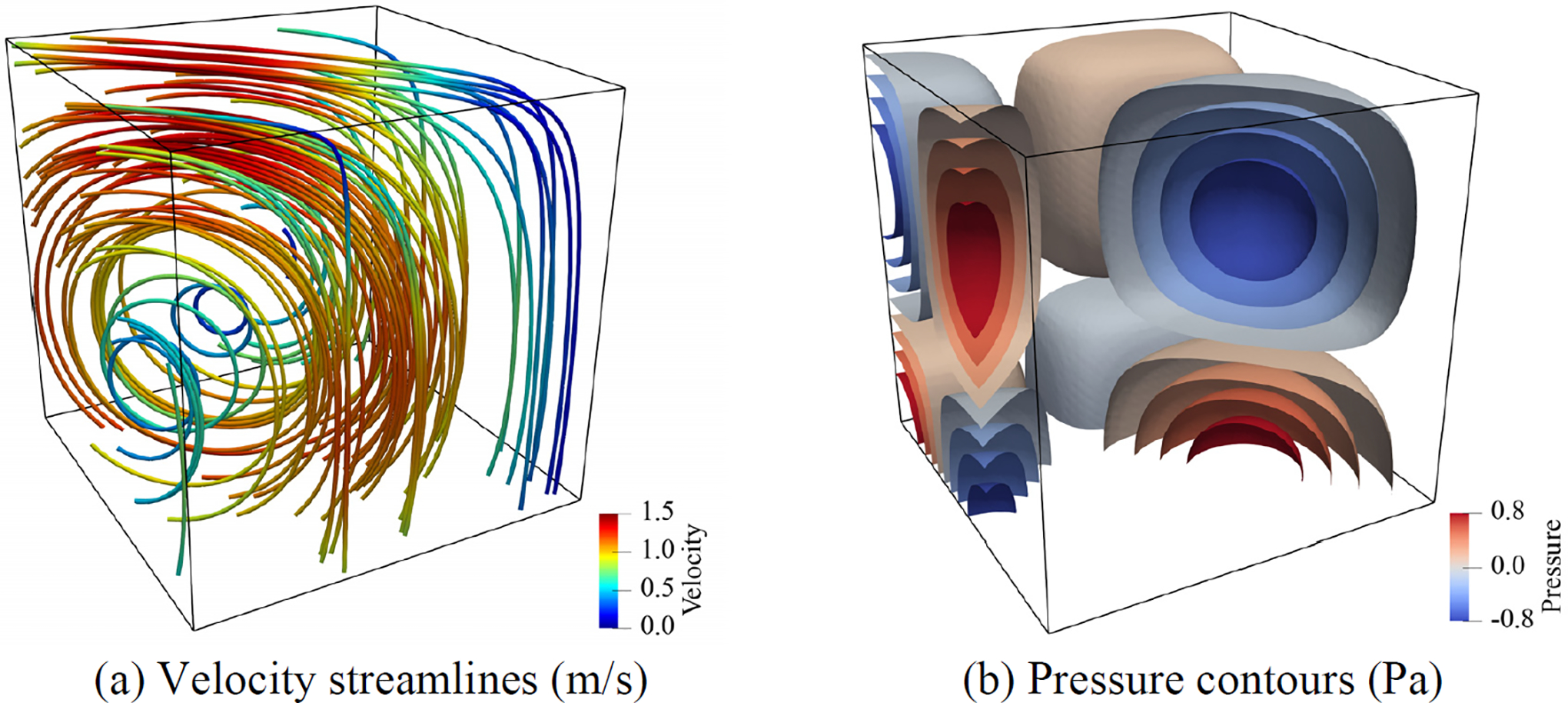
Computed streamlines and pressure contours in the domain -0.5≤x,y,z≤0.25.

**Figure 6.**
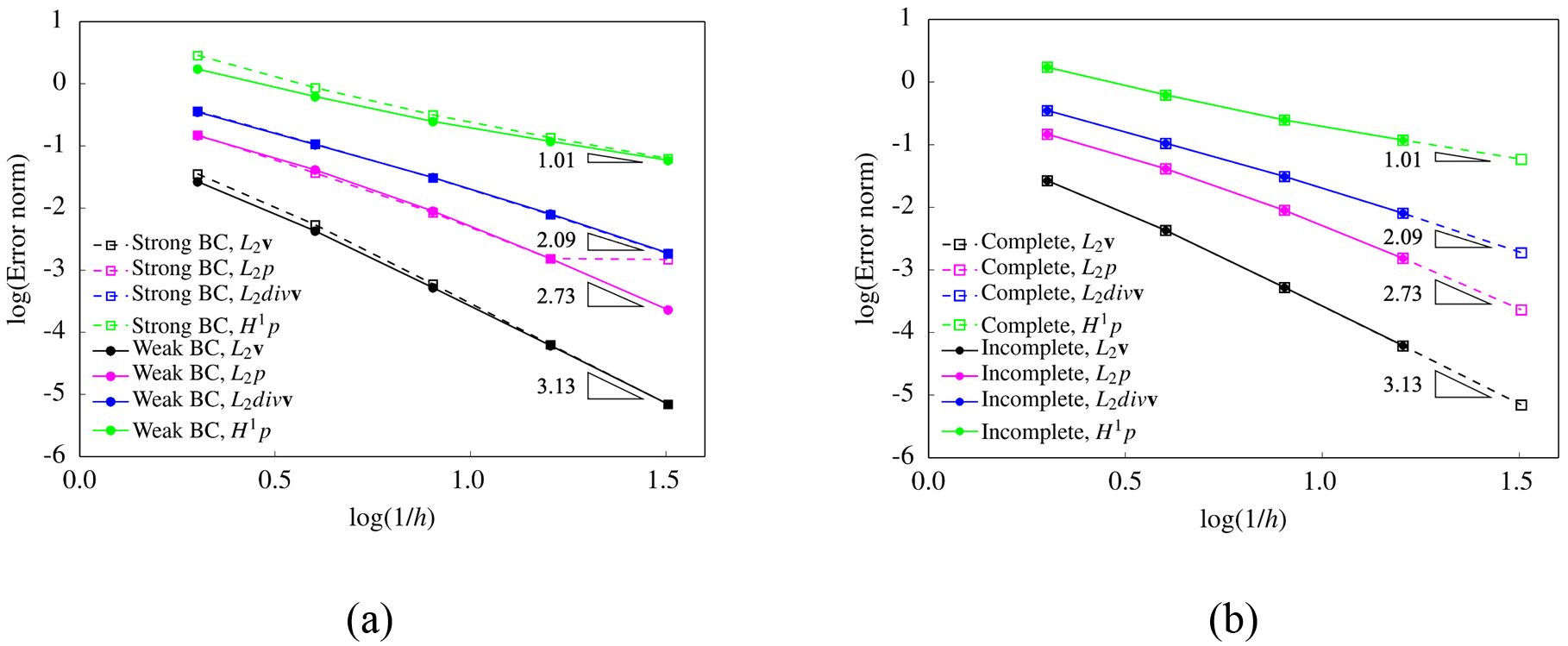
Rate of convergence study: (a) comparison between the strongly and weakly imposed boundary conditions, (b) comparison between the complete and the incomplete formulations.

We now develop a harder test case for the convergence-rate study presented in Section 4.1.1. We alter the computational domain by cutting the original domain right through the vortex such that -0.5≤x,y,z≤0.25. Since exact solution for this problem exists, which is given in Eq (36), the boundary conditions at this bounding surface can be applied both strongly as well as weakly. It is important to realize that in this problem where the domain boundary cuts through the vortex structure as shown in Figure 5, imposing the boundary conditions becomes more involved. Figure 6(a) compares the convergence rates for the strongly and weakly imposed boundary conditions. We observe that for the case of strongly imposed boundary conditions the $L_{2}$-norm of error for the pressure field does not converge for the finest mesh, while the errors for the weakly imposed boundary conditions uniformly converge for all the meshes. Figure 6(b) compares the convergence properties between the complete formulation Eq (33) and the incomplete formulation that is obtained by deactivating the boundary term that accounts for the viscosity gradient, i.e., the fourth boundary term in Eq (33). We realize that not including the boundary term with the shear-rate effects leads to catastrophic divergence of the method for the finest mesh, while keeping this term in the formulation leads to uniform convergence all through the mesh refinement. This numerical study justifies the crucial importance of this term in practical applications with non-Newtonian shear-rate fluids.

### 4.2. Lid-driven cavity flow

Steady lid-driven cavity flow is chosen to verify the spatial accuracy of the formulation presented in Eq (33) for both Newtonian and non-Newtonian fluids. We compare the results with the benchmark data [15, 26–28] and with the numerical results for the strongly imposed boundary conditions.

**Figure 7.**
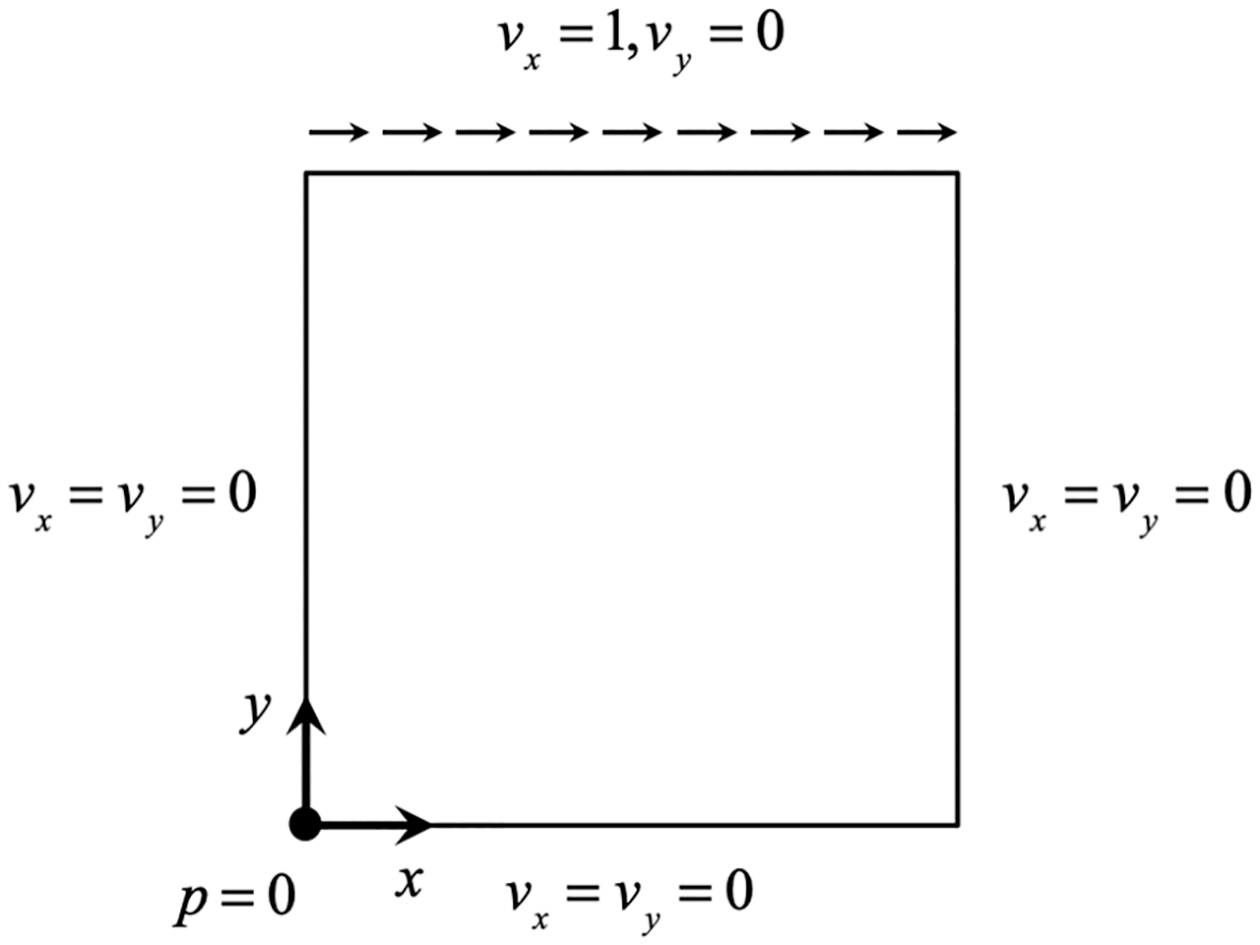
Spatial configuration and boundary conditions for the steady lid-driven cavity flow.

Figure 7 shows the biunit domain with the Dirichlet boundary conditions for the velocity field applied along all the faces. A constant unit velocity U=1 is applied in the x-direction at the top surface while no-slip boundary condition is applied at the other three surfaces. The zero pressure condition is specified at the left bottom corner to eliminate the arbitrary constant. We test both the Newtonian and the shear-thinning models as presented below.

#### 4.2.1. Newtonian fluids

For Newtonian fluids, Reynolds number is defined based on the lid-velocity U and domain width L. We consider three cases of Reynolds number Re=1000,5000 by setting the density ρ=1 and the viscosity $\mu =10^{-3},2\times 10^{-4}$, respectively. The computational domain is discretized using uniformly distributed N×N (where N=40,80) quadrilateral elements.

**Figure 8.**
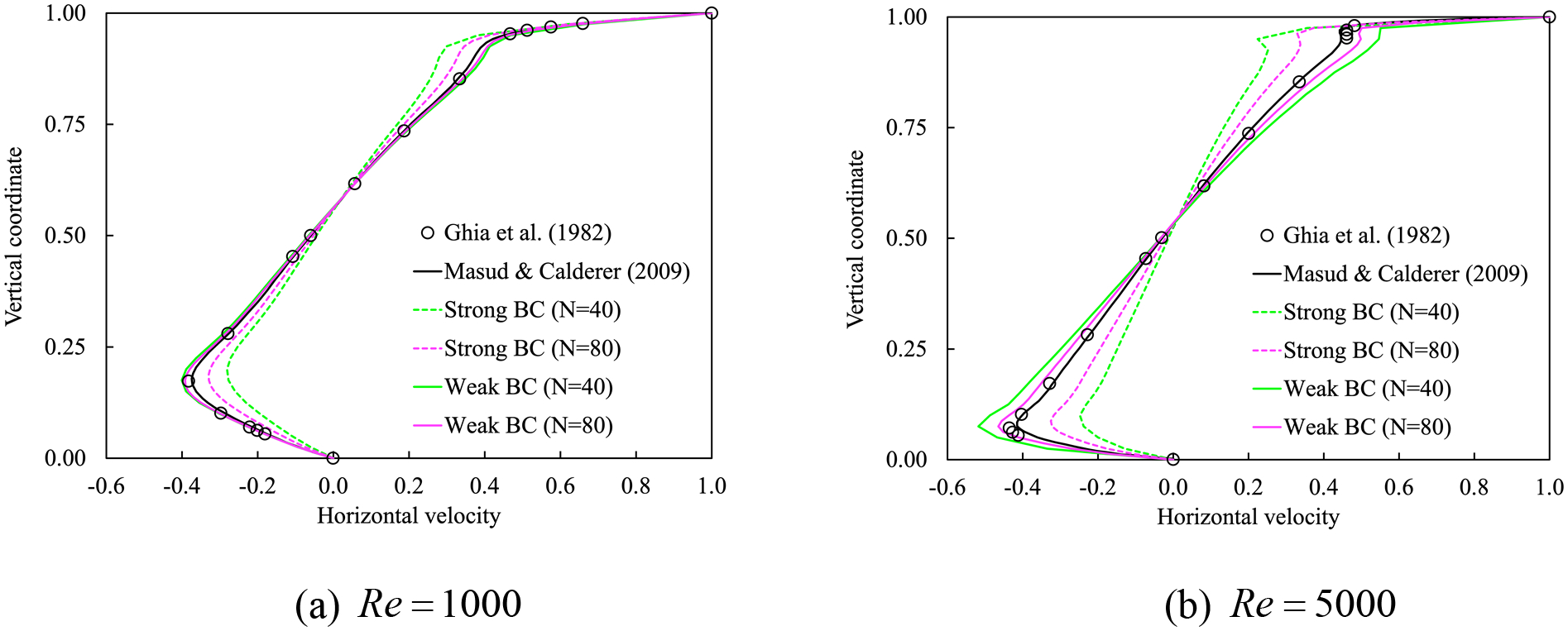
Horizontal velocity along the vertical line passing through the center for the lid-driven cavity flow with Newtonian fluids.

Figure 8 presents the profiles of the horizontal velocity along the vertical center line at x=0.5. We compare the computed results of strongly and weakly imposed boundary conditions with the reference results that are computed on the stretched meshes [26,26]. We observe that the weakly imposed boundary conditions successfully capture the reference profile and they outperform the strongly imposed boundary conditions on all the meshes.

#### 4.2.2. Shear-thinning non-Newtonian fluids

In this section, we employ the power-law and the Carreau-Yasuda models given in Eqs (6) and (7), respectively, for the shear-thinning fluids. The Reynolds number for the power-law fluids is defined by $Re=\rho U^{2-n}L^{n}/\mu$, as given in reference [15]. We set the exponent n=0.5, the density ρ=1, and the viscosity parameter $\mu =2\times 10^{-3}$, which yields Re=500. The material parameters for Carreau-Yasuda fluids are set equal to $\mu_{\infty }=0.00345,\mu_{0}=0.056,\lambda =1.902,n=0.22,a=1.25$, and ρ=1, which yields the Reynolds number Re=290 based on $\mu_{\infty }$. We employ (i) stretched, and (ii) uniform quadrilateral meshes, where the number of elements along an edge is N=40,80,160 for the power-law model, and N=20,40,80 for the Carreau-Yasuda model.

**Remark**: *The power-law model gives rise to singularity when* $\dot{\gamma }\approx 0$. *To keep the viscosity bounded at very low shear-rates, we impose a lower cap on the shear-rate*, $\dot{\gamma }\geq \varepsilon$, *where we have employed* $\varepsilon =10^{-16}$. *This treatment is equivalent to applying an upper bound for the viscosity*, $\eta (\dot{\gamma })\leq \eta (\varepsilon )$. *For the shear-thinning power-law fluids in* Section 4.2.2, *the viscosity is bounded by* $\eta (\dot{\gamma })\leq 200,000$.

**Figure 9.**
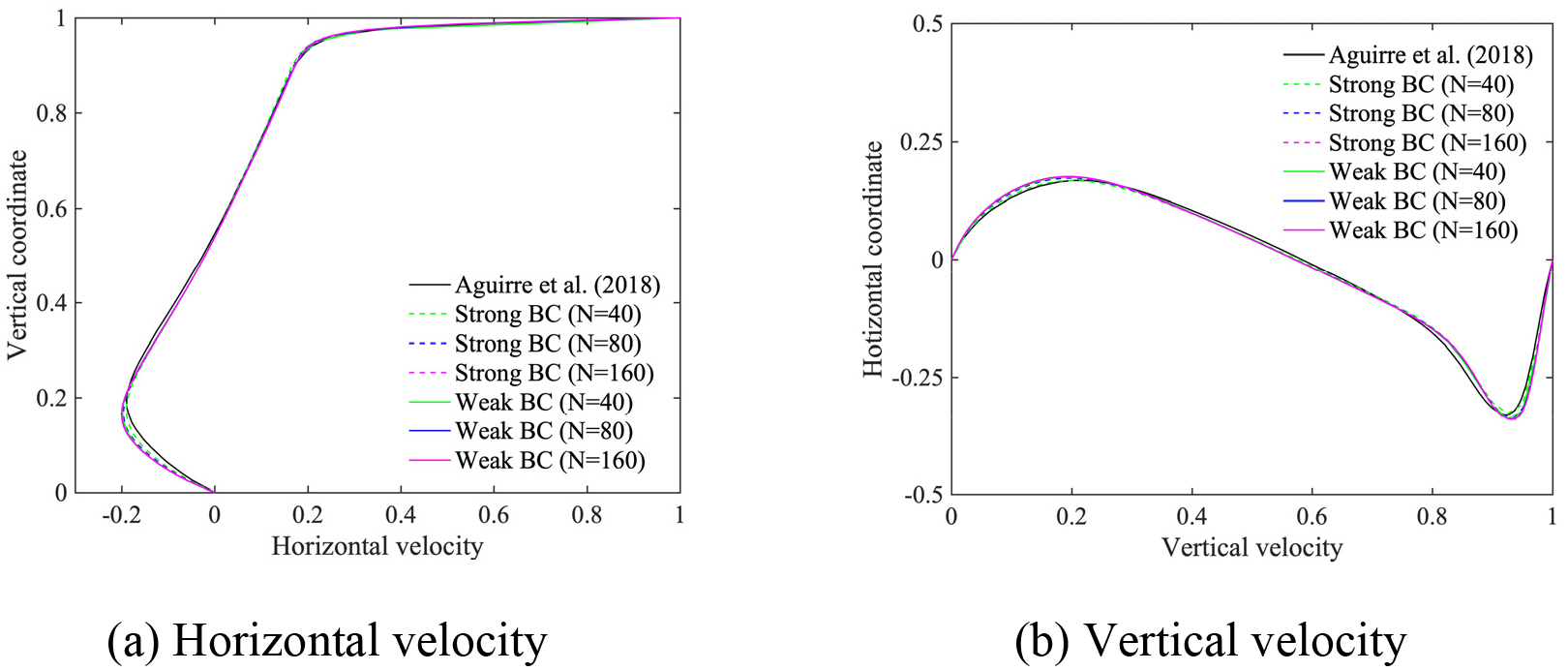
Horizontal and vertical velocity along the vertical and horizontal center line for the lid-driven cavity flow with the power-law model on the stretched meshes.

**Figure 10.**
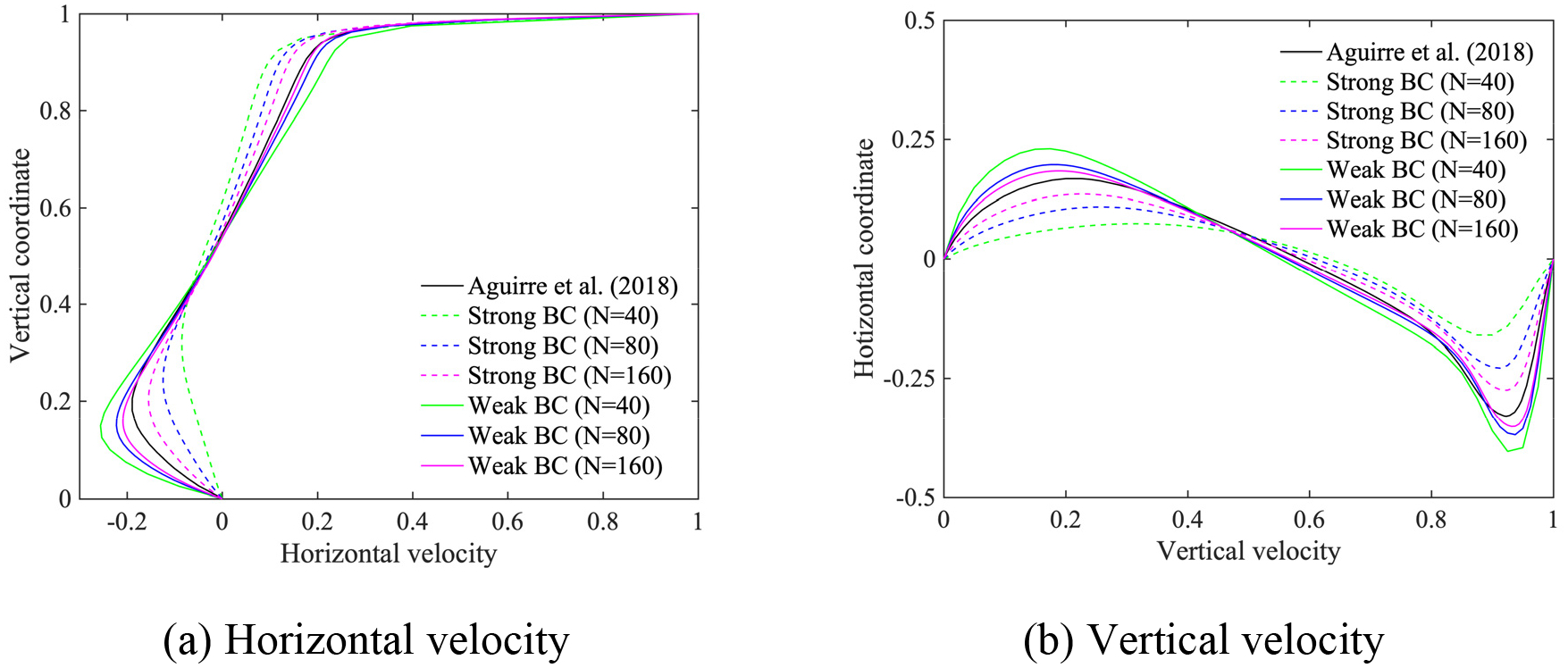
Horizontal and vertical velocity along the vertical and horizontal center line for the lid-driven cavity flow with the power-law model on the uniform meshes.

**Figure 11.**
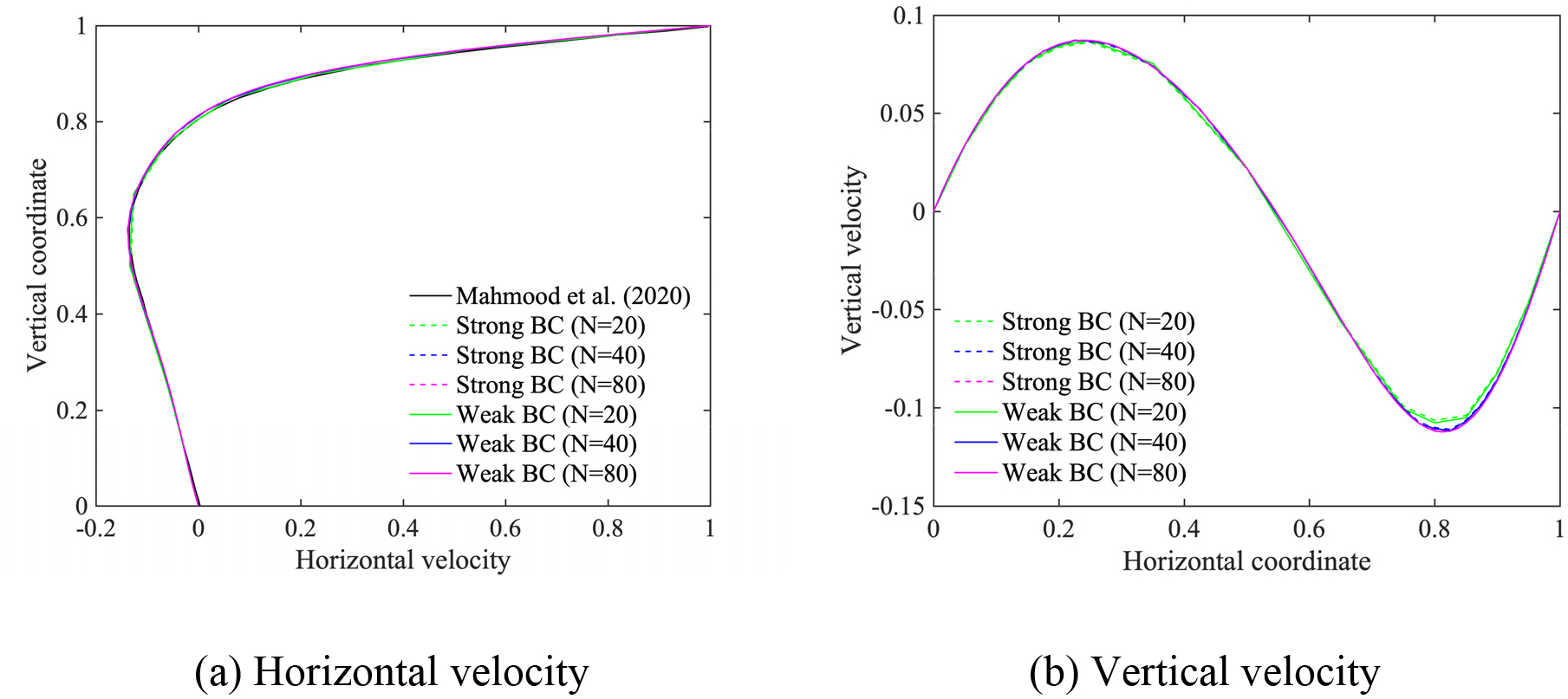
Horizontal and vertical velocity along the vertical and horizontal center line for the lid-driven cavity flow with the Carreau-Yasuda model on the stretched meshes.

**Figure 12.**
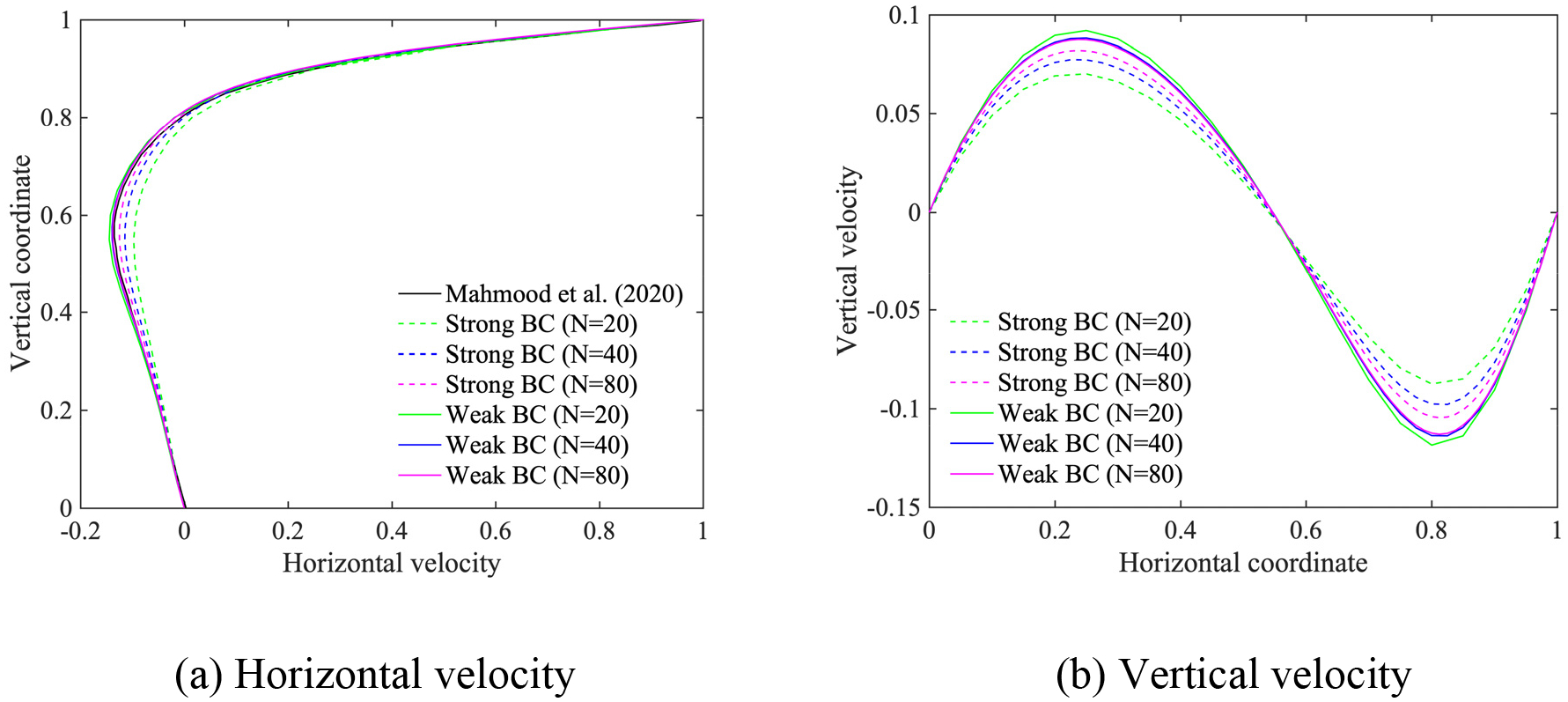
Horizontal and vertical velocity along the vertical and horizontal center line for the lid-driven cavity flow with the Carreau-Yasuda model on the uniform meshes.

Figures 9–12 present the comparison of the computed velocity profiles on the stretched and on the uniform meshes with the reference results [15, 28]. The reference results for the power-law and the Carreau-Yasuda models are computed on 180 × 180 and 128 × 128 meshes, respectively. We achieve good comparative results on meshes of different resolution for the power-law and Carreau-Yasuda models which indicates that the proposed method can handle different non-Newtonian constitutive equations. For the case of stretched meshes, all mesh configurations yield very accurate results in the boundary layer region, as shown in Figures 9 and 11. For the case of uniform meshes, the numerical results for both strongly and weakly imposed boundary conditions approach the reference results as a function of mesh refinement, as shown in Figures 10 and 12. However, the weakly imposed boundary conditions result in higher accuracy in the boundary layer region as compared to the strongly imposed boundary conditions. This feature demonstrates the enhanced mathematical attributes of the weakly imposed boundary conditions on the uniform meshes that may not have optimal mesh resolution for the boundary-layer flows.

**Figure 13.**
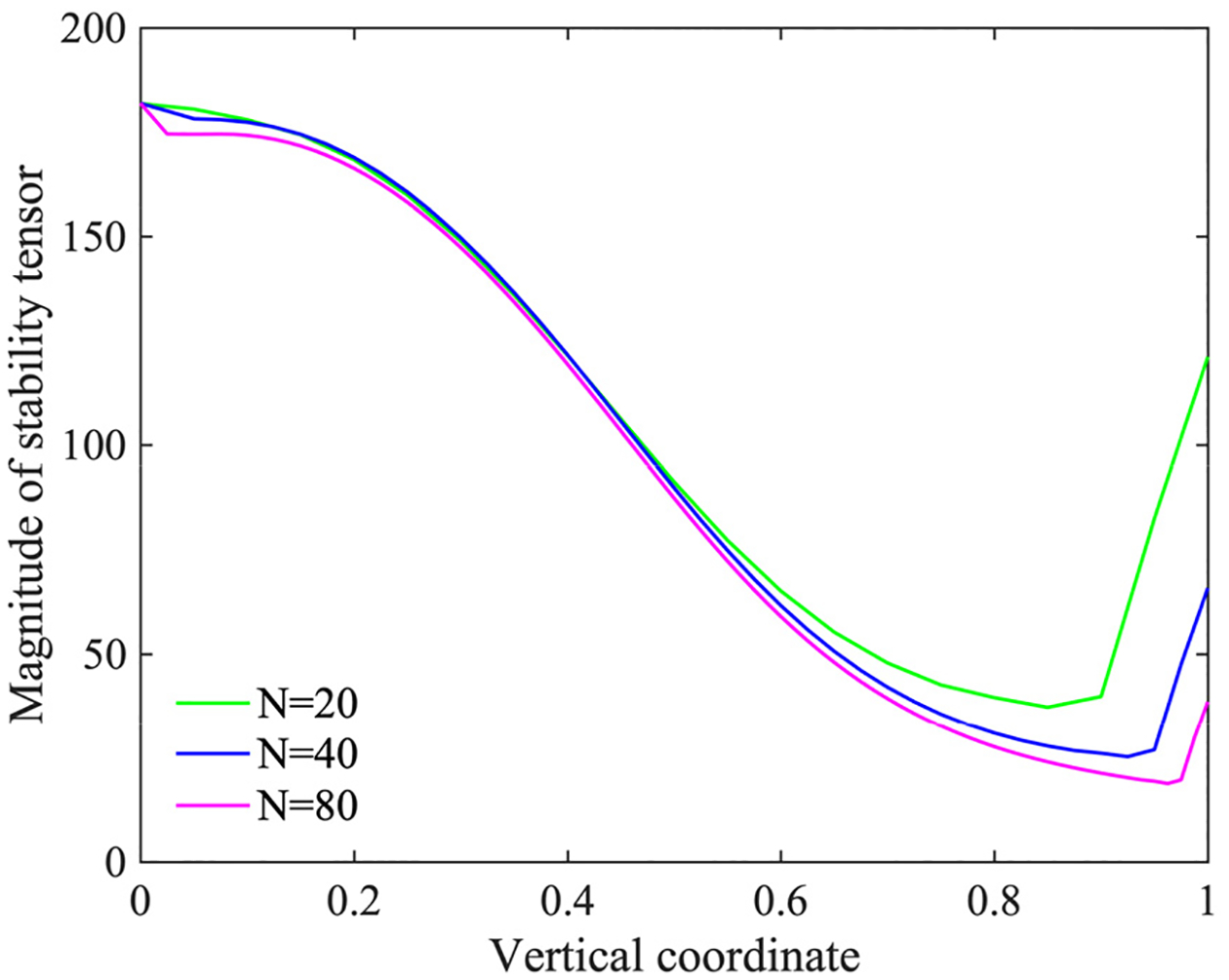
The magnitude of the non-dimensionalized stabilization tensor $∥\tau_{g}∥h/\mu_{\infty }$ along the vertical boundary at x=1.

Figure 13 shows the magnitude of the non-dimensionalized stabilization tensor $∥\tau_{g}∥h/\mu_{\infty }$ computed on the uniform meshes along the left vertical boundary for the case of Carreau-Yasuda model. The stabilization tensor $\tau_{g}$ defined in Eq (28) is calculated over the layer of elements adjacent to the boundary $\Gamma_{g}$. The magnitude of the tensor is computed as $∥\tau_{g}∥=\sqrt{\tau_{g}:\tau_{g}},h$ is the length of the element edge, and $\mu_{\infty }$ is the asymptotic viscosity at the infinite shear-rate in Carreau-Yasuda model. As the meshes are refined, the width of the spatial domain comprising the first layer of elements adjacent to $\Gamma_{g}$ reduces, thereby asymptoting to the dimension $n_{\text{sd}}-1$. Figure 13 shows that all the meshes yield similar spatial distribution of the non-dimensional stability parameter and they gradually converge to the results from the finest mesh that yields a better representation of the notion of the edge.

### 4.3. Flow in a curved tube

**Figure 14.**
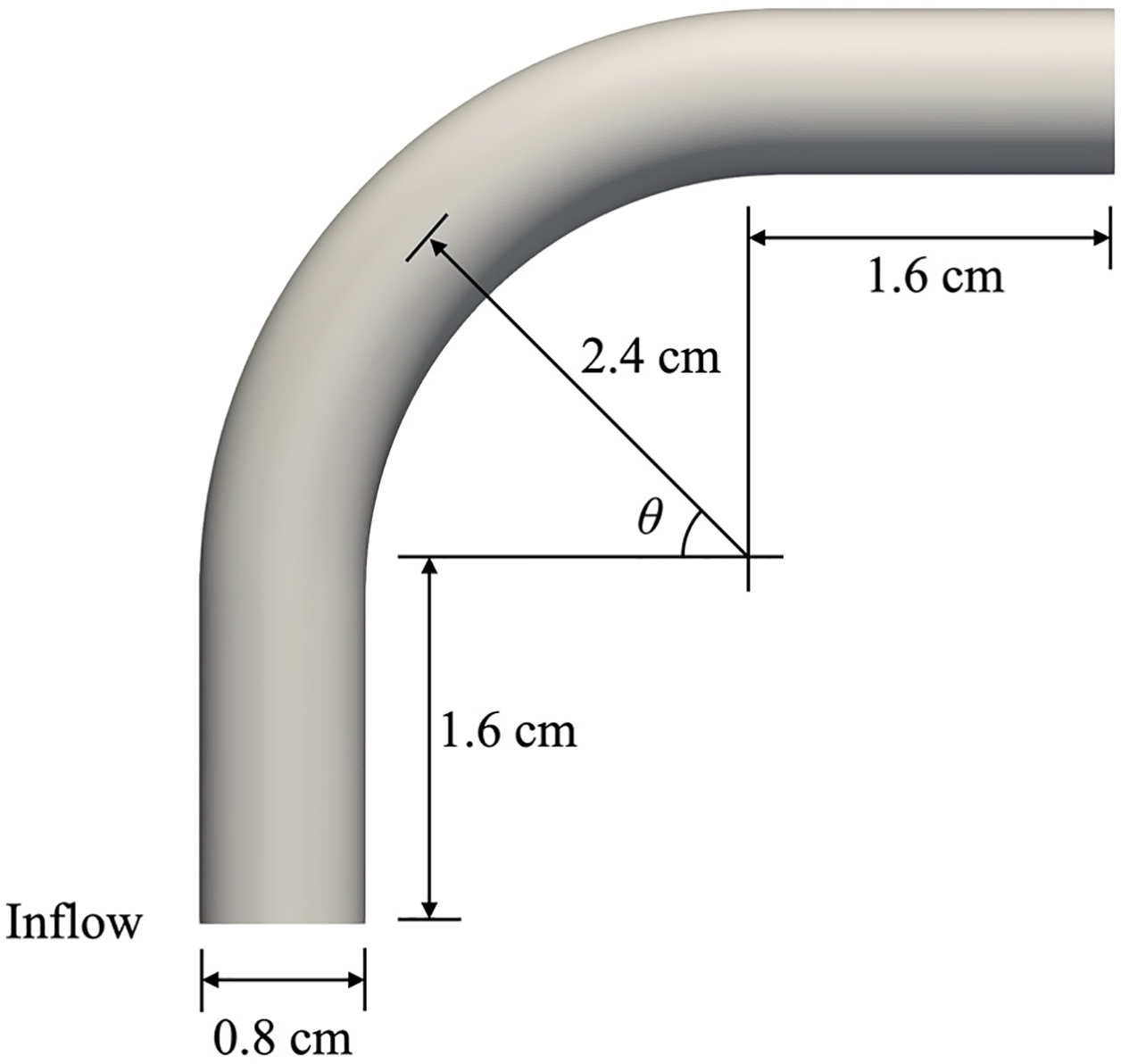
Geometry and dimensions of the curved tube.

This test case is a model for flow in an idealized femoral artery [29] where we investigate both Newtonian and non-Newtonian constitutive equations. The geometry of the curved tube and its dimensions are shown in Figure 14. The diameter of the tube is D=0.8 cm, the length of the straight segment is L=1.6 cm, the radius of curvature is R=2.4 cm, and the angle from the inlet is $\theta =0\sim 90^{\circ }$. The parabolic inflow velocity profile is prescribed at the bottom surface. The no-slip boundary condition is strongly or weakly imposed over the cylindrical surface, and the traction-free condition is applied at the outflow surface. We employ two successively refined quadratic tetrahedral meshes where the refined mesh is generated by reducing the element size by half. The description of the two meshes is given in Table 2.

#### 4.3.1. Verification of the method with the Newtonian fluid model

We first test our method with Newtonian fluids and compare the computed results with the experimental data [29] that was obtained via laser-Doppler velocity measurements. The density of fluid is $\rho =0.8028\text{ g}/{\text{cm}}^{3}$ and the dynamic viscosity is μ=0.1 dyne ⋅s/cm^2^. The average velocity at the inlet is U=109 cm/s, which yields Reynolds number Re=ρUD/μ=700.

**Figure 15.**
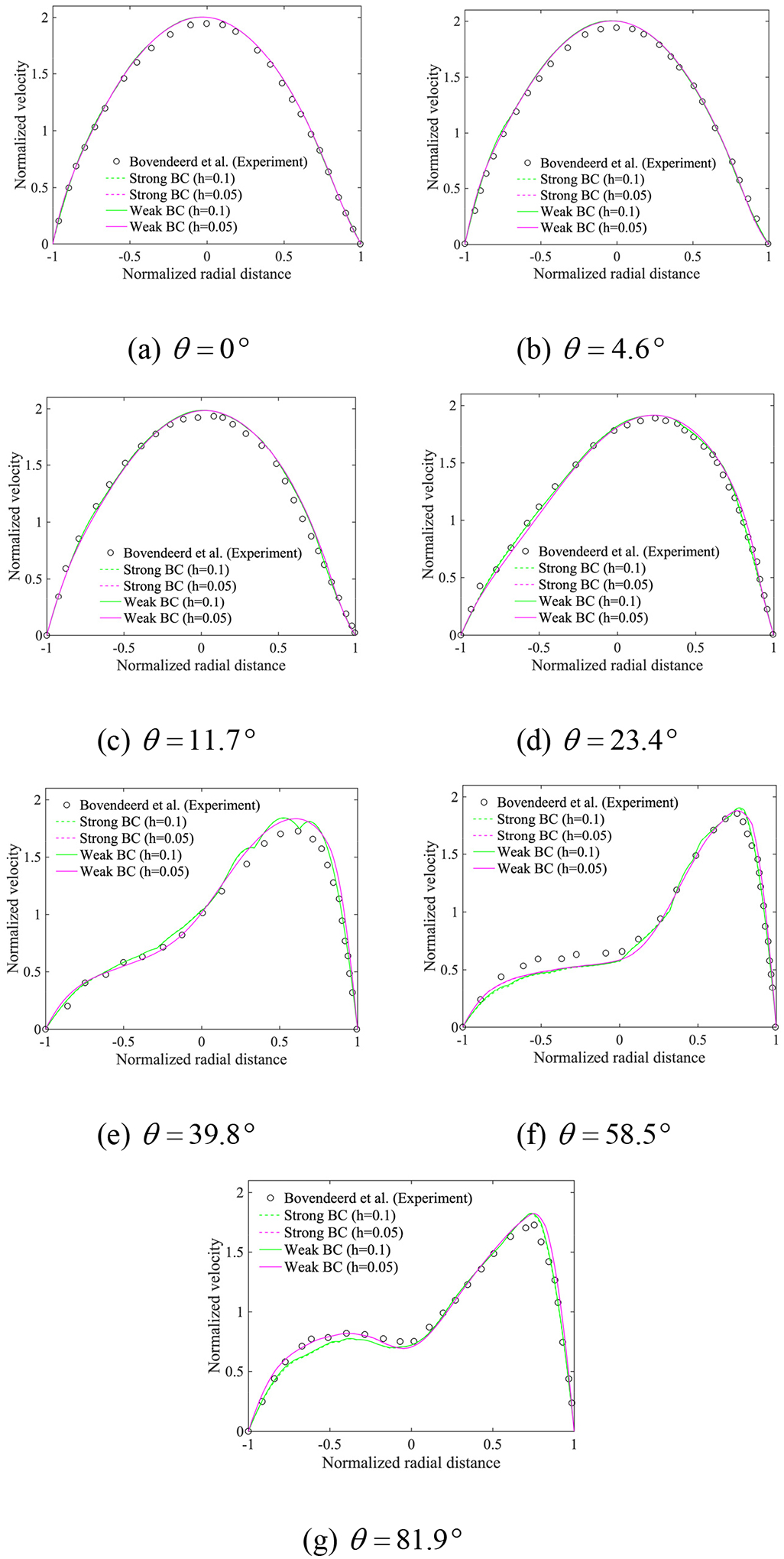
Profiles of the streamwise velocity across the cross-sections at various angles (The radial distance is normalized with respect to the radius of the tube, and the velocity is normalized with respect to the average velocity at the inlet).

Figure 15 presents the streamwise velocity along the cross sections subtended at various angles as shown in Figure 14. A good agreement between the computed results and experimental data is attained on the fine mesh. The coarse mesh has only eight quadratic elements along the diameter, with no-slip condition at the ends of the diameter, thereby giving rise to boundary layers at either end. Despite being relatively crude, the mesh is able to capture the profile quite accurately and this is attributed to the fine-scale modeling feature of the method. We also observe that both strongly and weakly imposed boundary conditions produce similar results on each mesh.

#### 4.3.2. Shear-thinning non-Newtonian blood flow

**Figure 16.**
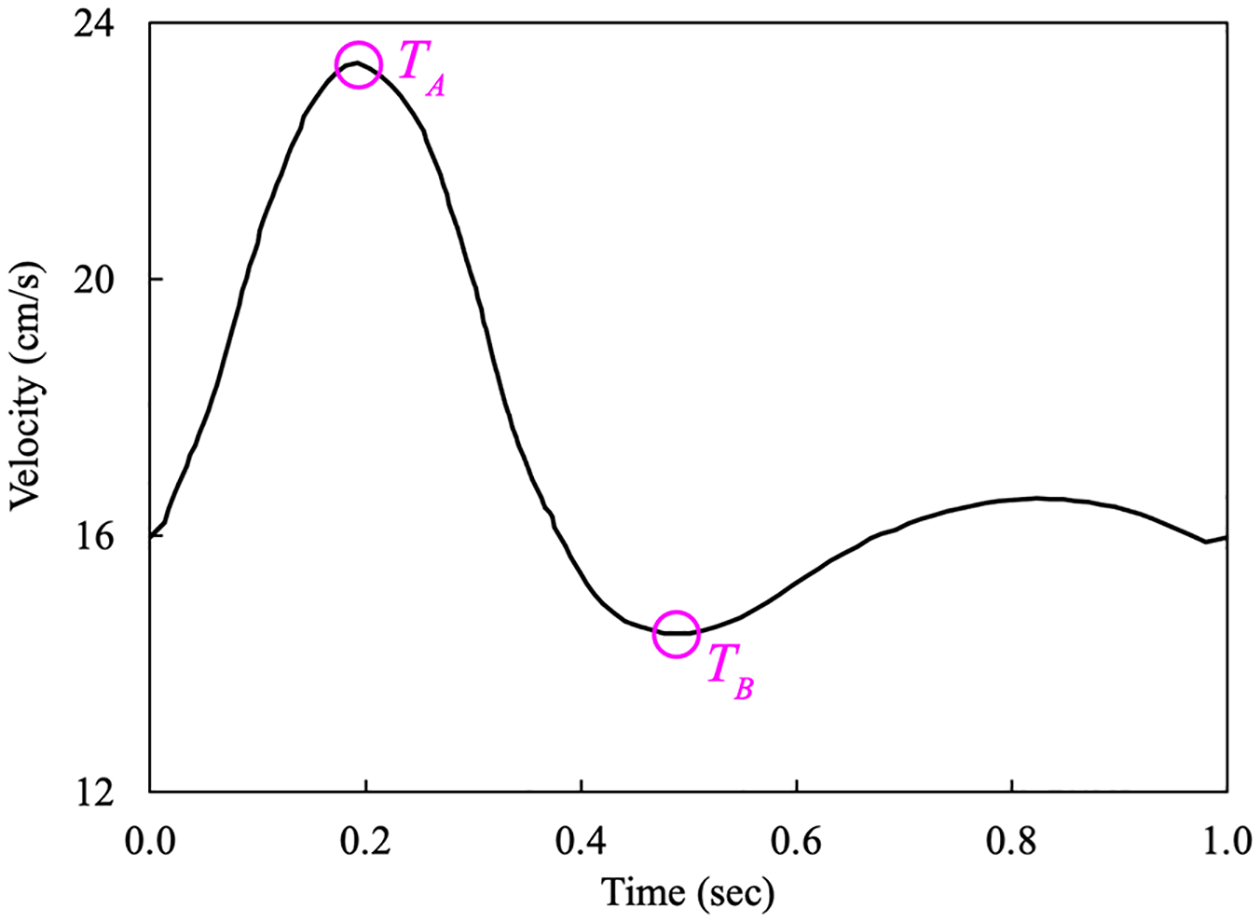
The velocity waveform applied at the center of the inflow surface (Time points $T_{A}$ and $T_{B}$ correspond to the maximum and minimum inflow velocity).

In this section, we consider pulsatility of blood flow in addition to the shear-thinning effects of blood. We employ the Carreau-Yasuda model in Eq (7) with the coefficients given in Table 3 that are taken from [13]. The density of blood is $\rho =1.06\text{ g}/{\text{cm}}^{3}$. The prescribed timevarying velocity at the center of the inlet is presented in Figure 16. We simulate three cardiac cycles with a time step size of Δt=0.01 s and take the numerical results from the last cycle for comparative study.

**Figure 17.**
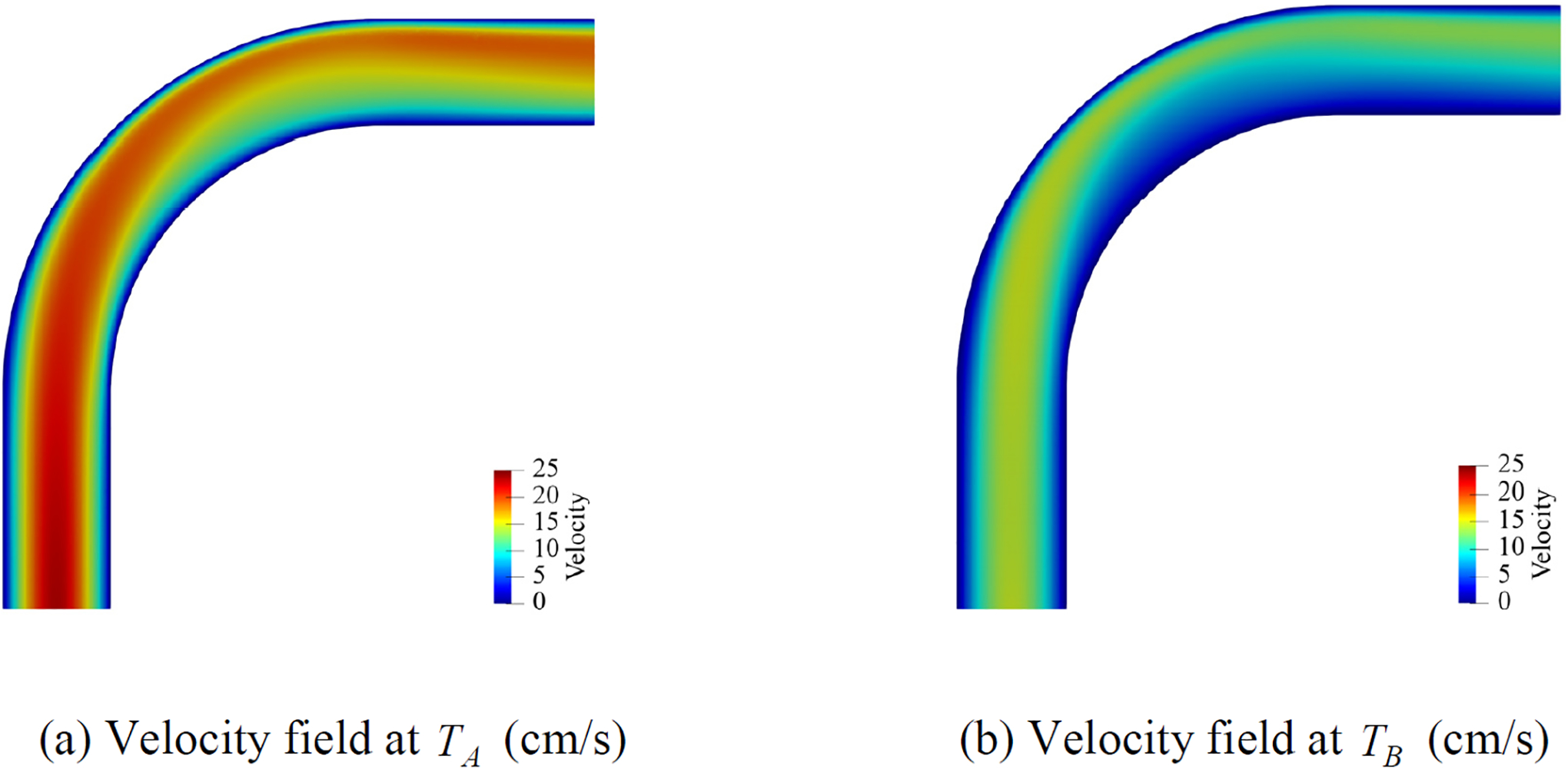
Numerical results for the velocity field on the fine mesh by weakly imposed no-slip condition.

**Figure 18.**
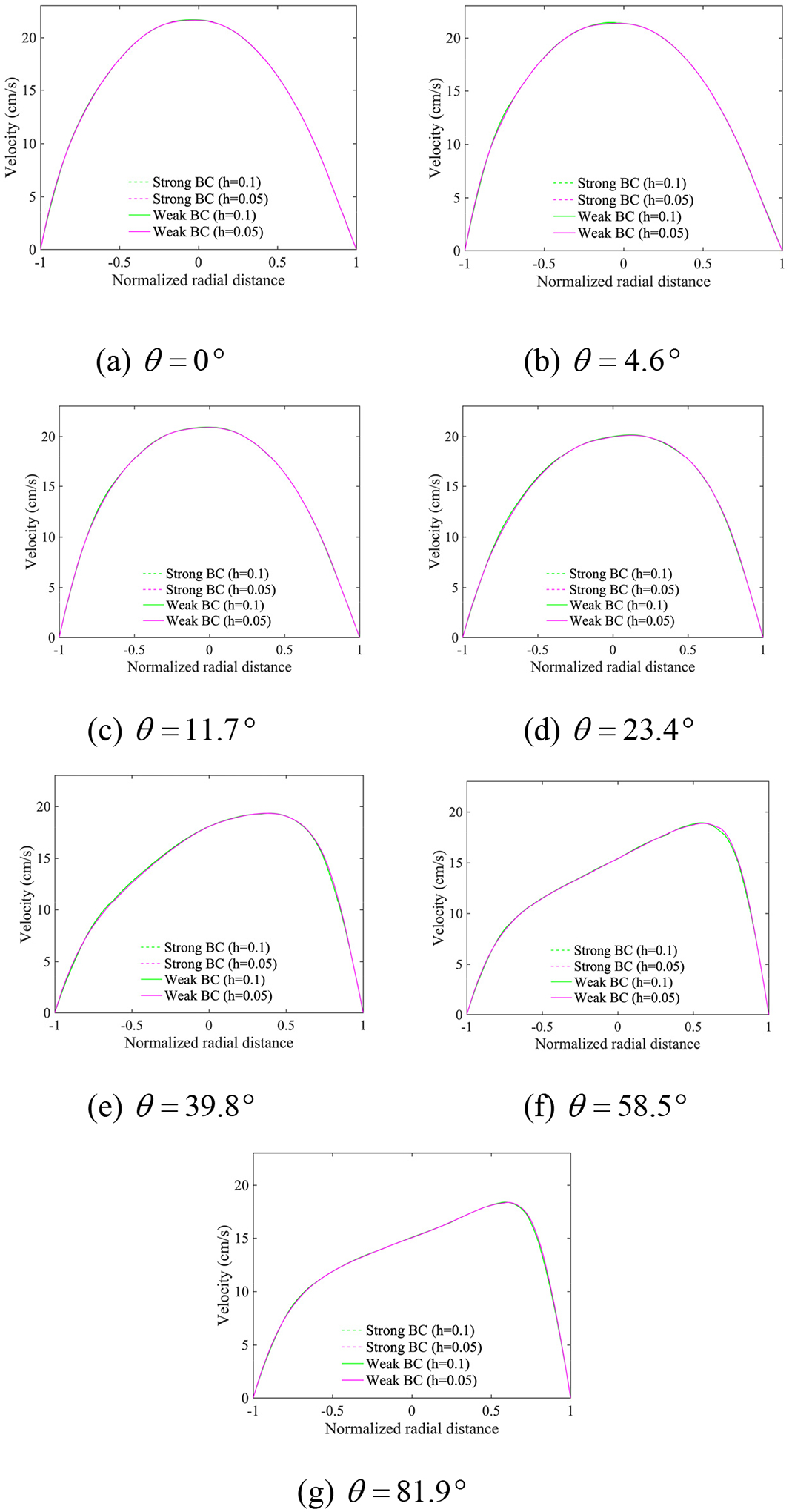
Profiles of the streamwise velocity across the cross-sections at various angles at the time point $T_{A}$ where the inflow velocity is the maximum (The radial distance is normalized with respect to the radius of the tube).

**Figure 19.**
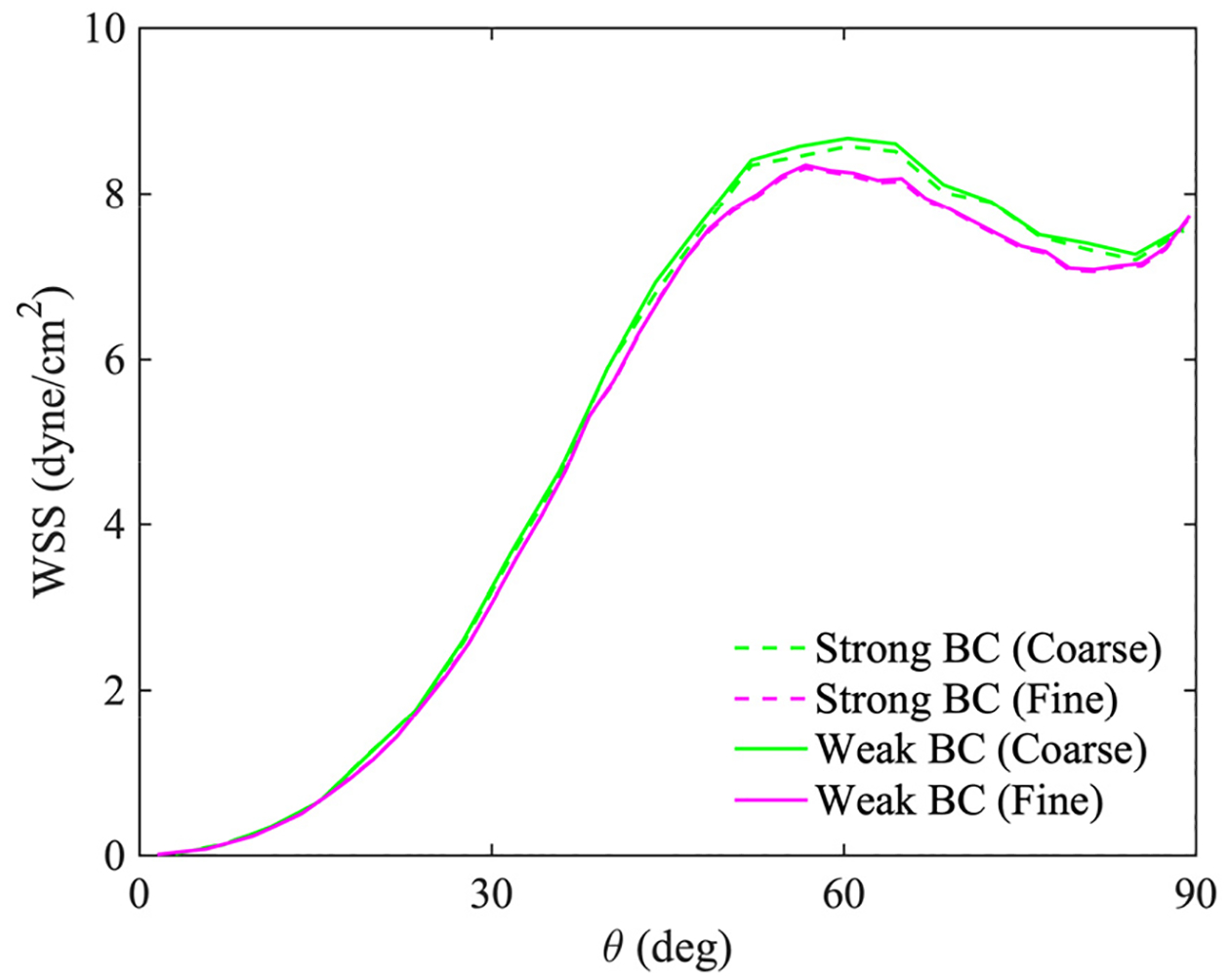
Time-averaged wall shear stress (WSS) for a cardiac cycle along the outermost circular curve in the bent tube.

Figure 17 presents the computed velocity field on the fine mesh by weakly imposing the no-slip condition at the wall. It shows that the boundary terms in Eq (33) accurately enforce the no-slip condition that leads to the boundary layer near the wall. Figure 18 compares the computed streamwise velocity at various angles between the strongly and weakly imposed boundary conditions. Figure 19 shows the wall shear stress (WSS) along the outermost curve of the bent tube. The WSS is averaged in time for a cardiac cycle. The comparisons of the velocity and WSS verify that both methods for imposing the essential boundary condition produce comparable results on the coarse and fine meshes. Table 4 summarizes the range of eigenvalues of the stabilization tensor τ for the case of weakly imposed boundary conditions. Since the eigenvalues remain positive, it shows that the stability tensor also stays bounded and positive-definite.

**Figure 20.**
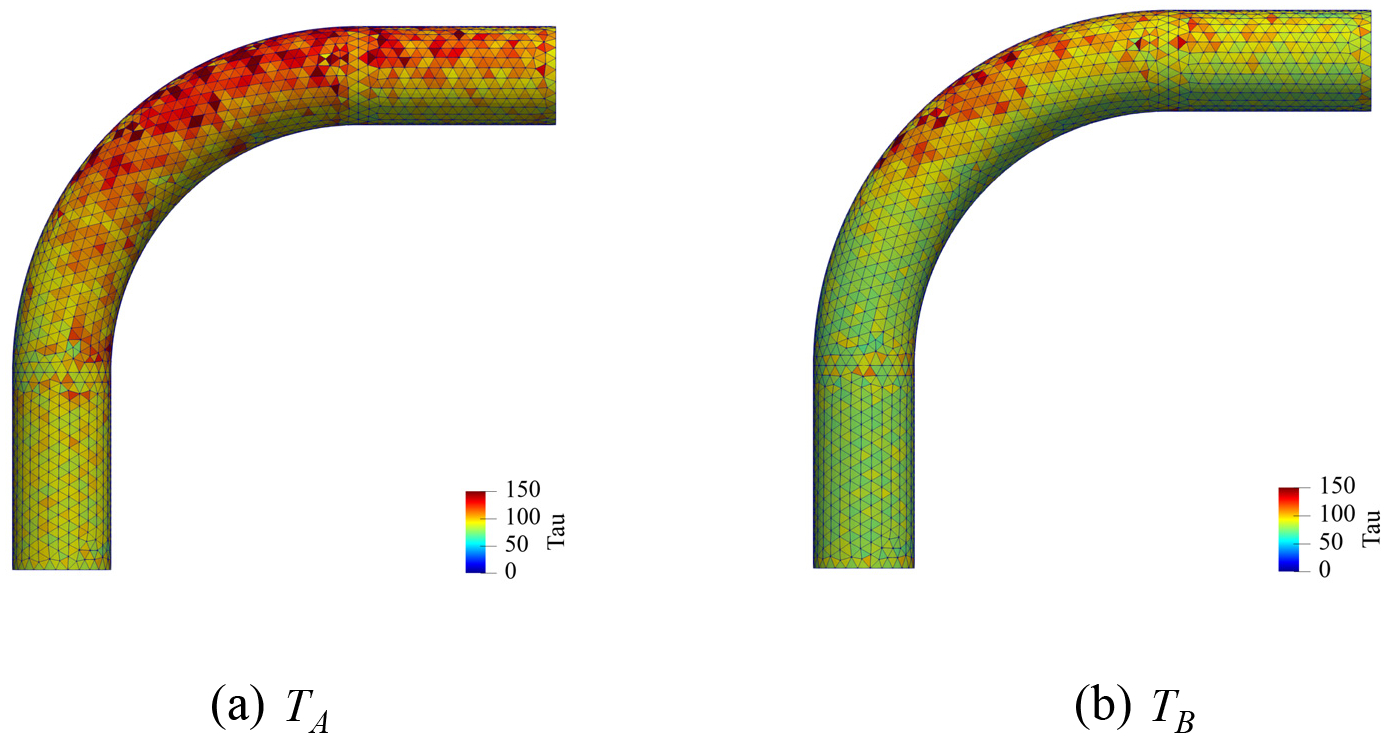
Spatial distribution of the magnitude of the non-dimensionalized stabilization tensor $∥\tau_{g}∥h/\mu_{\infty }$ computed on the intermediate mesh at two time points $T_{A}$ and $T_{B}$ indicated in Figure 16. The magnitude of the tensor is computed as $∥\tau_{g}∥=\sqrt{\tau_{g}:\tau_{g}},h$ is the element length-scale, and $\mu_{\infty }$ is the asymptotic viscosity at the infinite shear-rate in Carreau-Yasuda model.

A unique attribute of the proposed method is the variationally derived stabilization tensor $\tau_{g}$ that enforces the Dirichlet boundary condition. It is explicitly defined in Eq (28) and it results in element-wise optimal values for $\tau_{g}$ that are automatically calculated locally. Figure 20 displays the spatial distribution of the magnitude of the stabilization tensor $\tau_{g}$ computed at two time points $T_{A}$ and $T_{B}$ in a typical cardiac cycle. It shows that the value of $\tau_{g}$ tends to be larger where the velocity and its gradient are higher on the top surface and near the outlet. It also shows that the value of $\tau_{g}$ varies with time and is larger at the time point $T_{A}$ than at the time point $T_{B}$ because the inflow velocity is also higher at $T_{A}$.

### 4.4. Blood flow in a representative patient-specific arterial model

**Figure 21.**
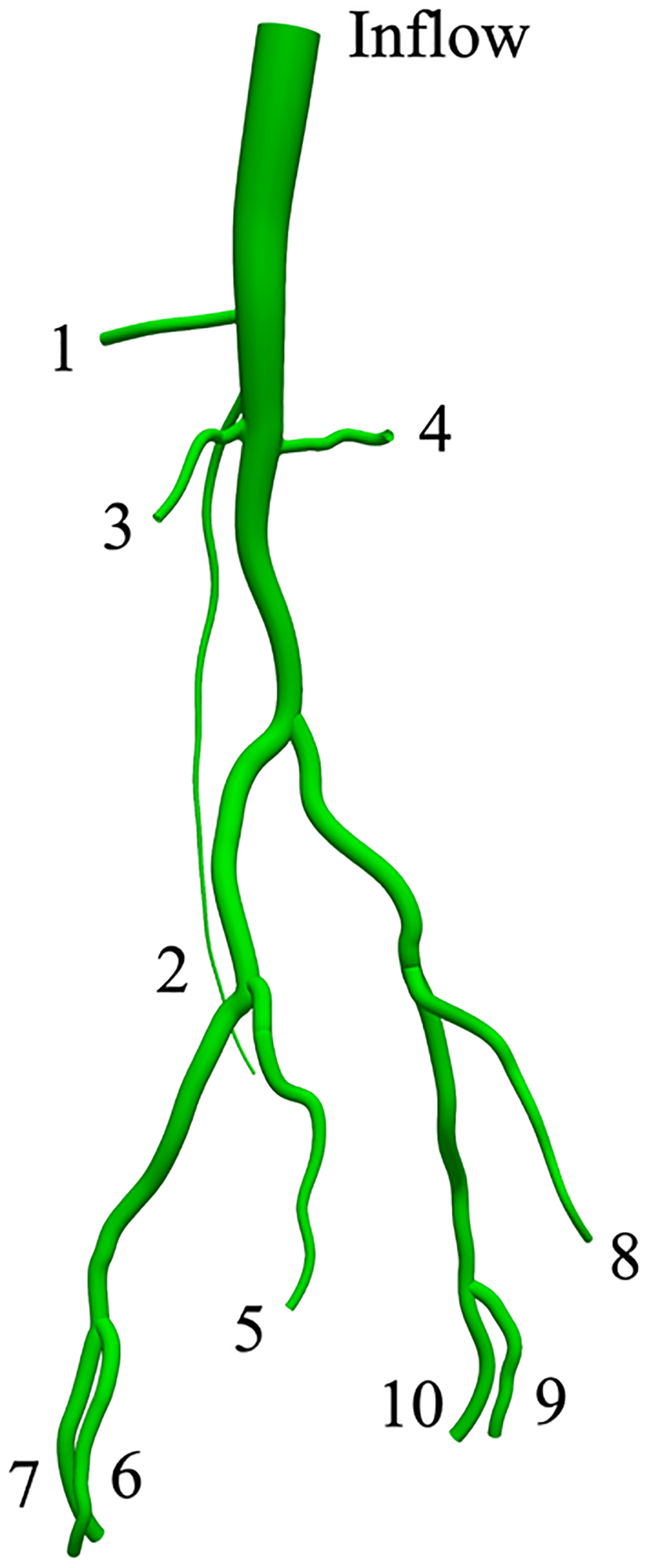
Geometric model of patient-specific arteries.

In this test case we take the method for weakly imposed boundary conditions to patient-specific aortic and femoral arteries of clinical relevance. We constructed a patient-specific geometric model using the cross-sectional CT scan images and the 3D geometry of the arterial tree branching from the thoracic aorta to the femoral arteries as shown in Figure 21. The geometric model was discretized using quadratic tetrahedral elements where the number of nodes and elements in the mesh are 586,116 and 388,810, respectively. We simulated three cardiac cycles with a time increment of Δt=0.005 s, which corresponds to 220 time steps during a typical cardiac cycle.

**Figure 22.**
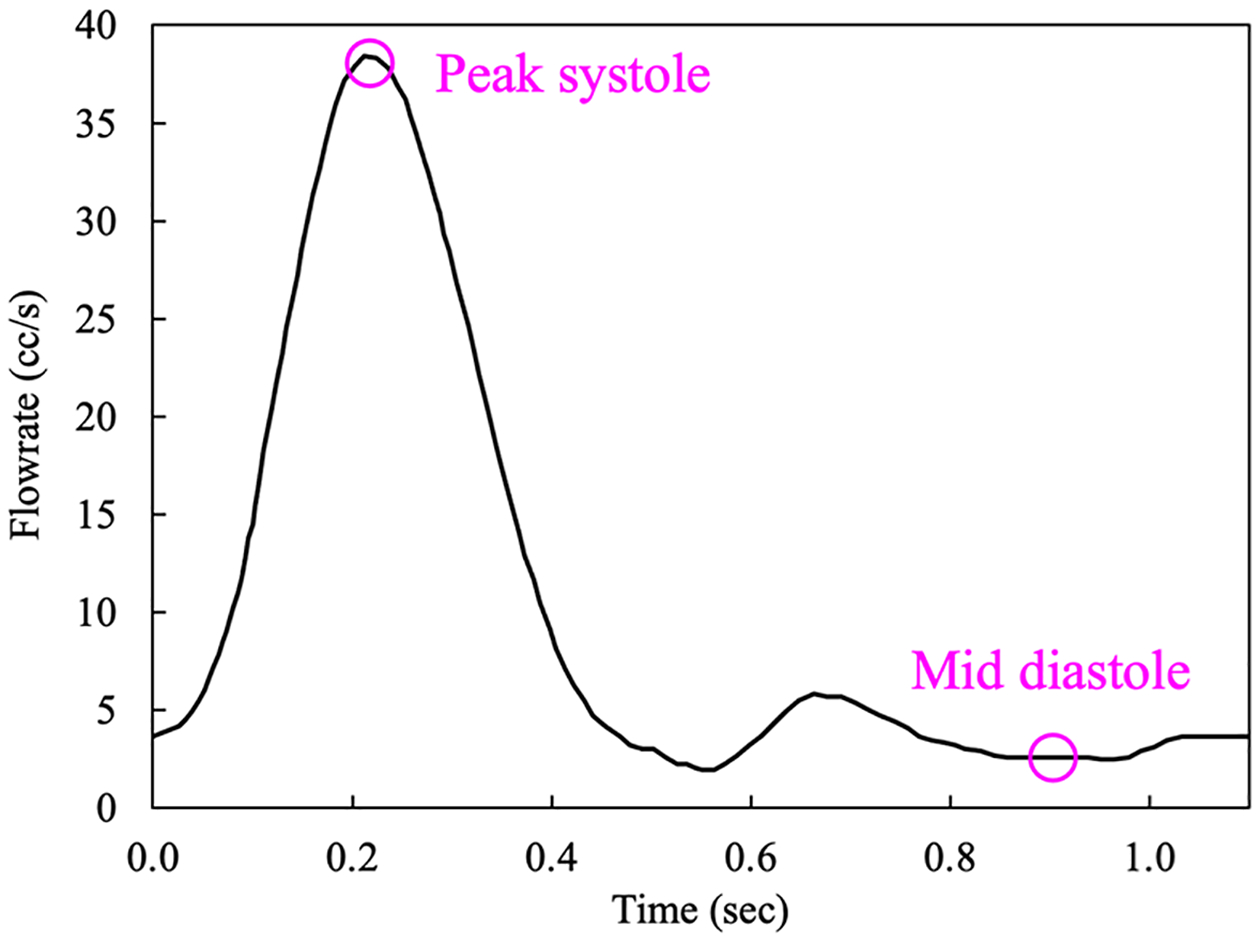
Flowrate at the inlet of the aortic artery

The non-Newtonian effects of blood are accounted for via the Carreau-Yasuda model with parameters given in Table 3. The density of blood is $\rho =1.06\text{ g}/{\text{cm}}^{3}$. The flowrate shown in Figure 22 is specified at the inflow surface of the thoracic artery by imposing the parabolic distribution of velocity. The artery wall is considered as no-slip surface. The resistance boundary condition in Eq (38) that produces physiological pressure waveform at the outlets is imposed at all the outlets to incorporate the downstream resistive effects of the arteries [17,30].

(38) $$p=R\int_{\Gamma_{\text{outflow}}}v\cdot n\;d\Gamma +p_{0}$$

where R is the resistance parameter and $p_{0}=80\text{ mmHg}$ is the constant downstream pressure. The resistance parameter for each branch is tuned based on the flow-rate in the branches, which is calculated with the traction-free conditions, to obtain a pressure amplitude between 80–110 mmHg at the outlets. Table 5 summarizes the value of the resistance parameter for each branch.

Non-physical backflow at the outlet is often encountered in cardiovascular simulations, and it can destabilize the solution. To address this issue, we apply backflow stabilization [31,32] to the outflow boundary surface by adding the following term to the left-hand side of Eq (33).


(39) $$B\left(w,v\right)=\{\begin{array}{ll} -{\left(w,\rho \left(v\cdot n\right)v\right)}_{\Gamma_{\text{ouflow }}} & \text{ if }v\cdot n<0\\ 0 & \text{ otherwise } \end{array}$$

This term is only activated where the velocity is pointed inwards to the fluid domain at the outflow surface.

**Remark**: *In this patient-specific test case, where no-slip condition is weakly imposed all over the arterial wall surface, the derived stabilization tensor* $\tau_{g}$ *defined in* Eq (28) *is multiplied by a constant α=20, which leads to better convergence in the non-linear solution*.

**Figure 23.**
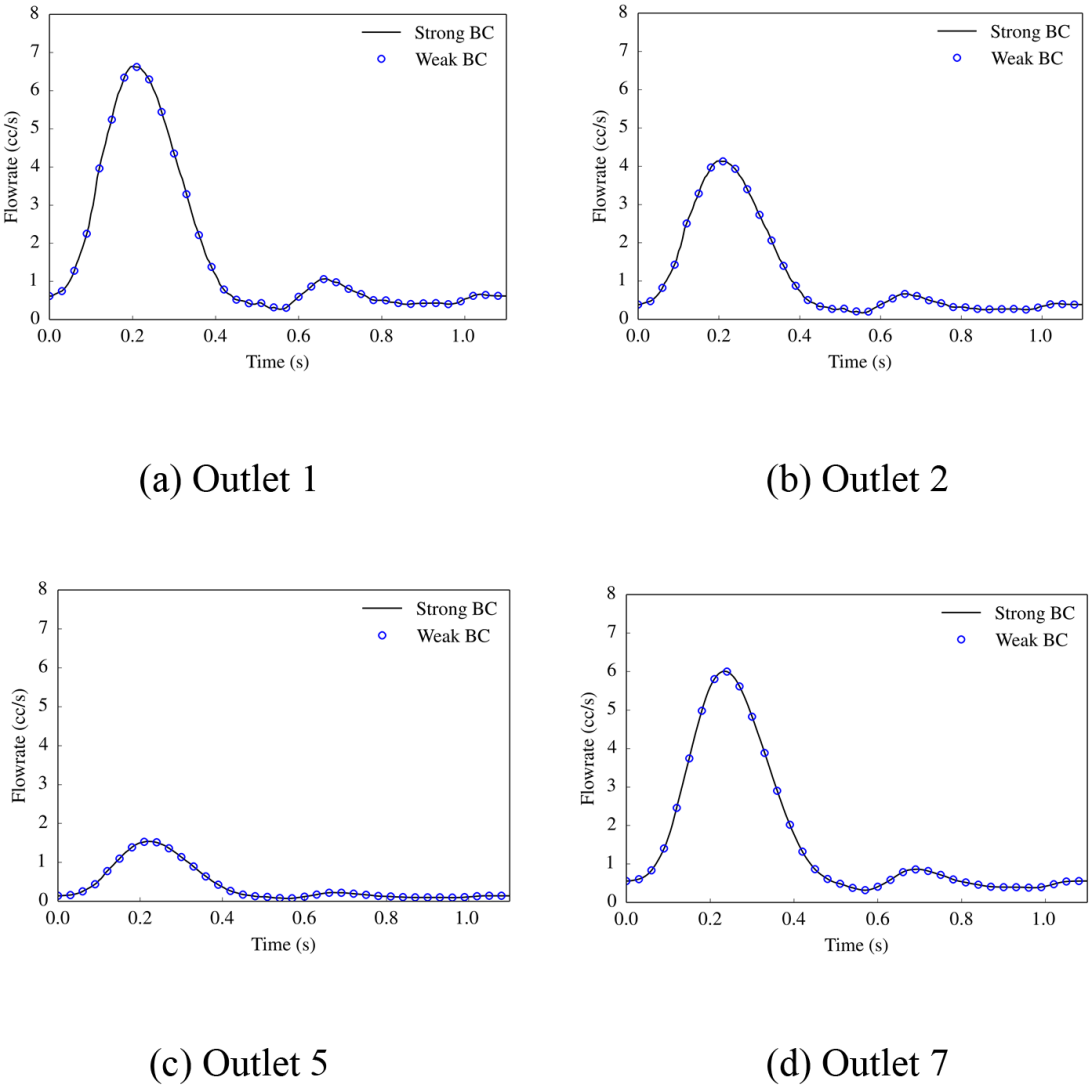
Comparison of the flowrate at the outlets (marked in Figure 21) between the strong and weak BC cases.

**Figure 24.**
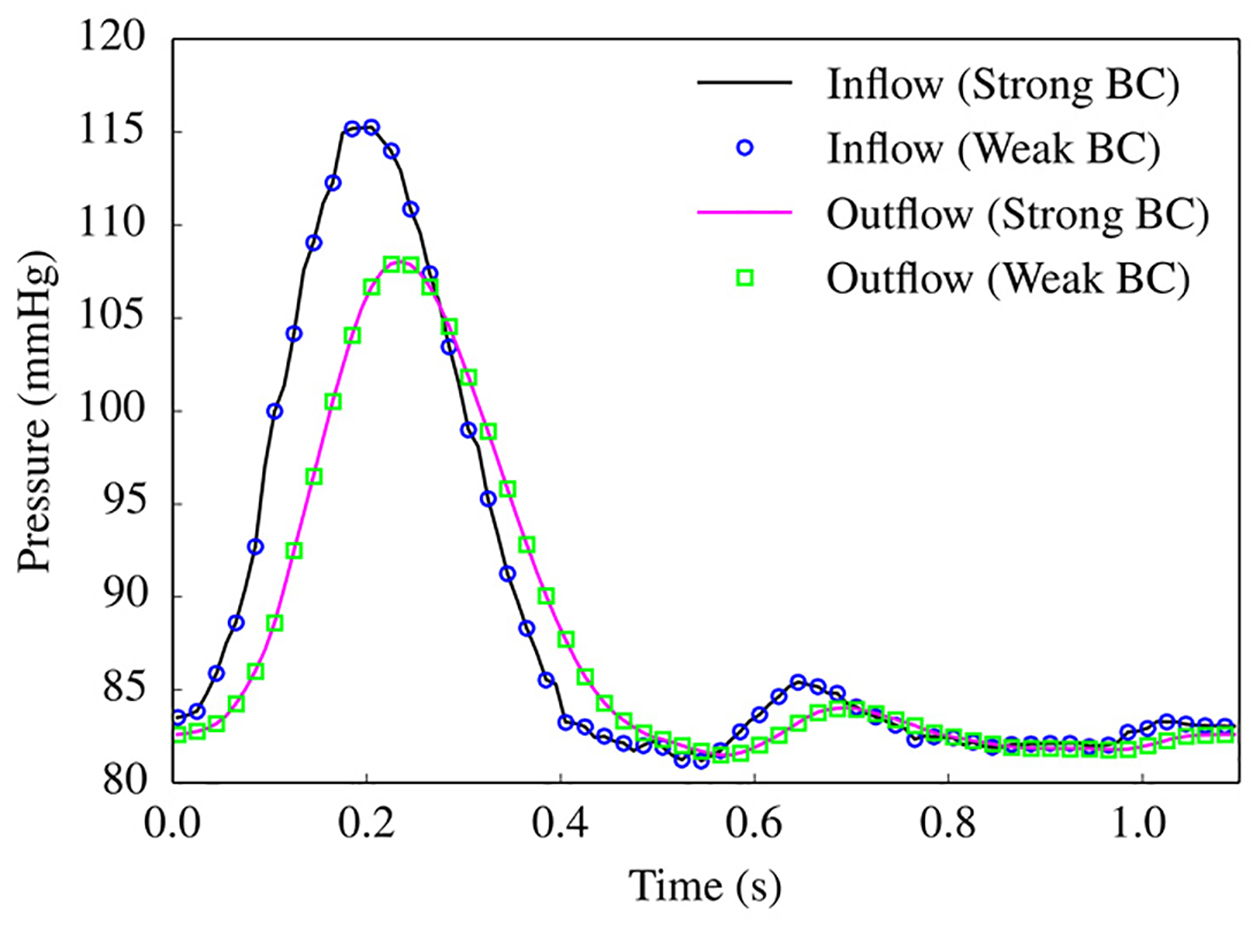
Comparison of the pressure at the inflow and outflow surfaces between the strongly and weakly imposed BC cases. (The outflow pressure is computed at outlet 7 marked in Figure 21).

Figure 23 compares the flowrate at the outlets of some representative branches for the cases of strongly and weakly imposed essential boundary conditions, and a good agreement between the computed solutions is observed. Figure 24 shows that the computed pressure profiles with the strong and weak imposition of boundary conditions closely match for the inflow boundary and the outflow boundary, respectively. This test case verifies that the weakly imposed boundary condition produces equivalent blood flow patterns in the arterial system as are produced by the strongly imposed boundary conditions.

**Figure 25.**
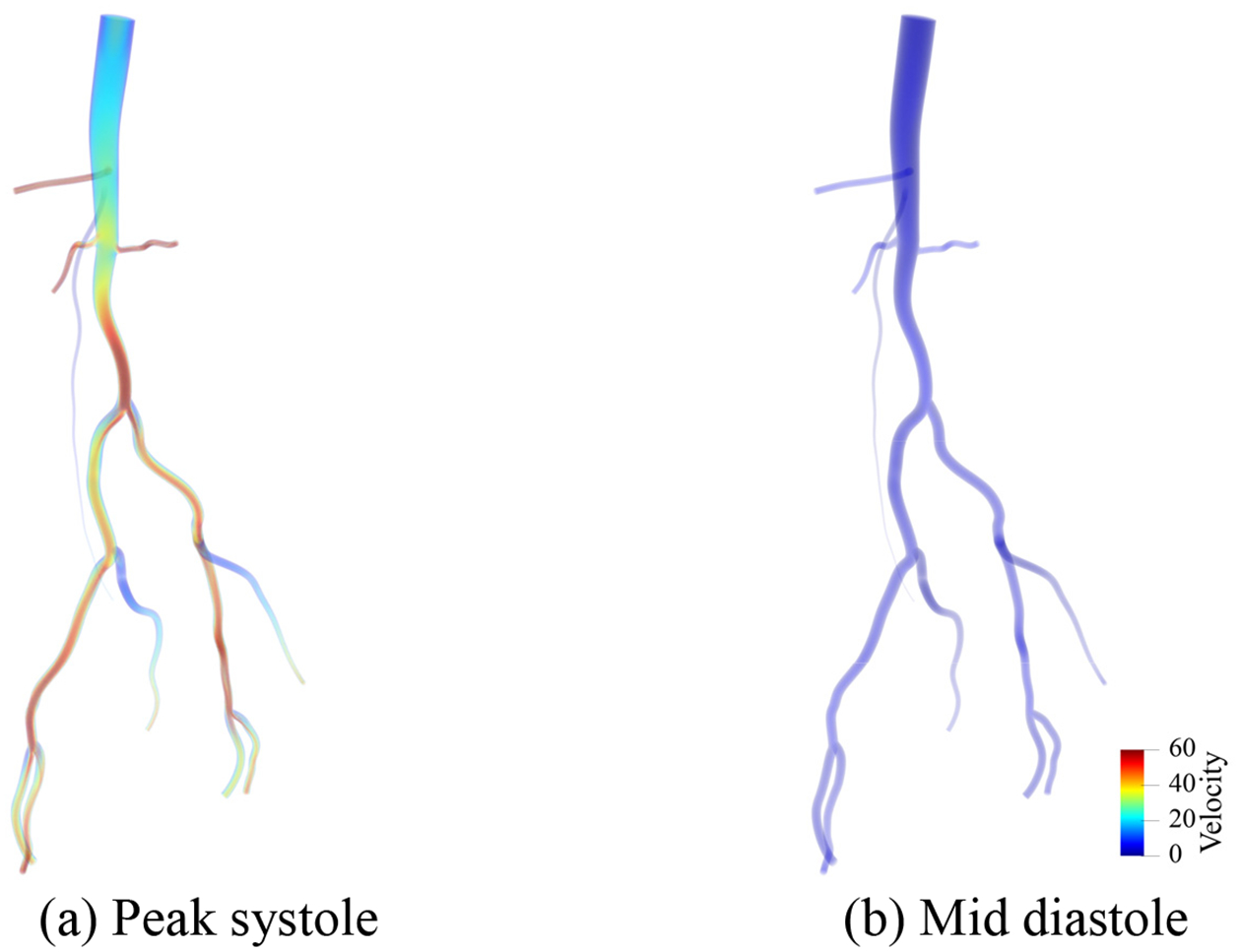
Volume rendering of the velocity magnitude computed via the weakly imposed boundary condition at the peak systole and the mid diastole. (Unit: cm/s).

**Figure 26.**
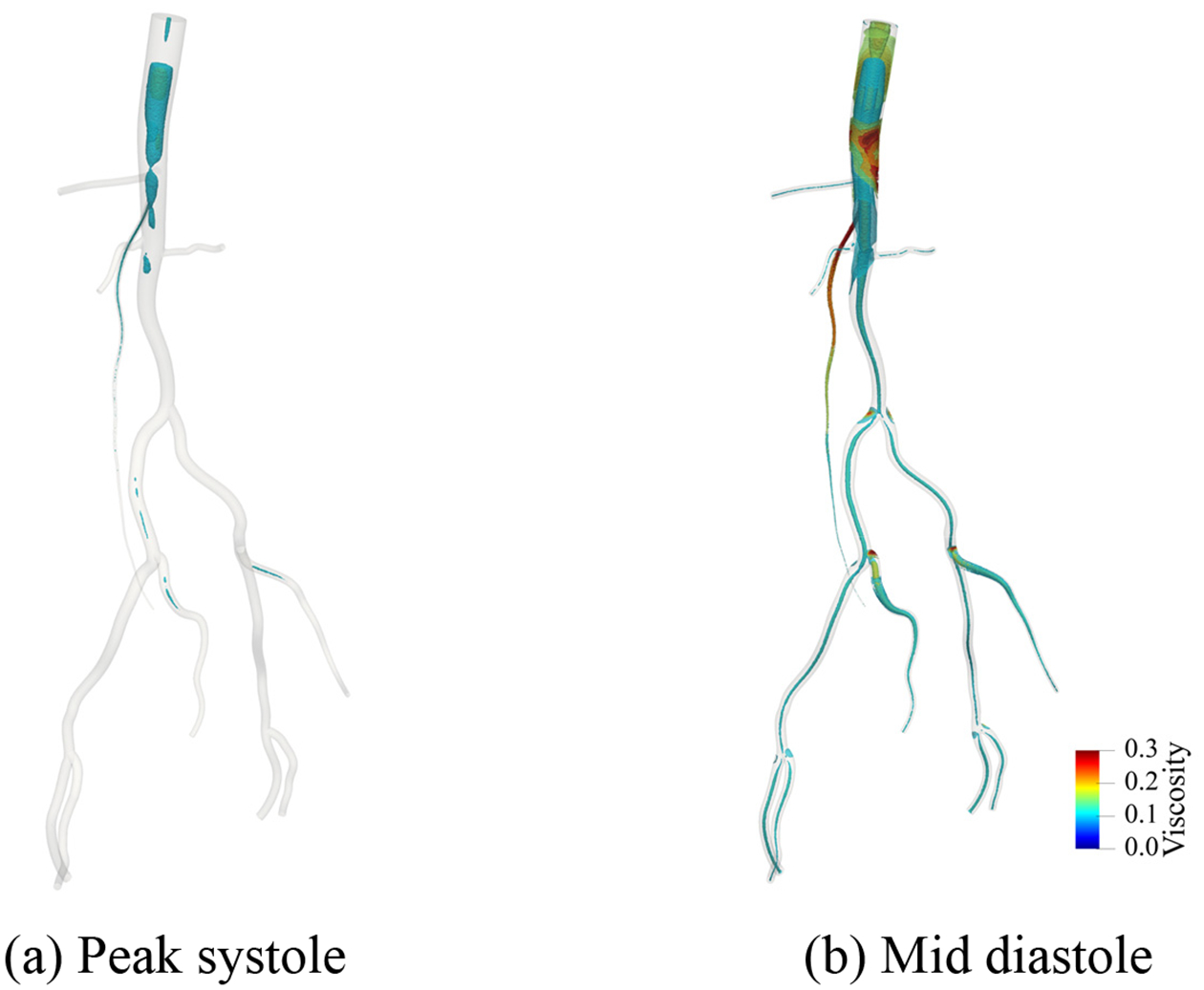
Viscosity contours computed via the weakly imposed boundary condition at the peak systole and the mid diastole. (Unit: dyne · s/cm^2^).

**Figure 27.**
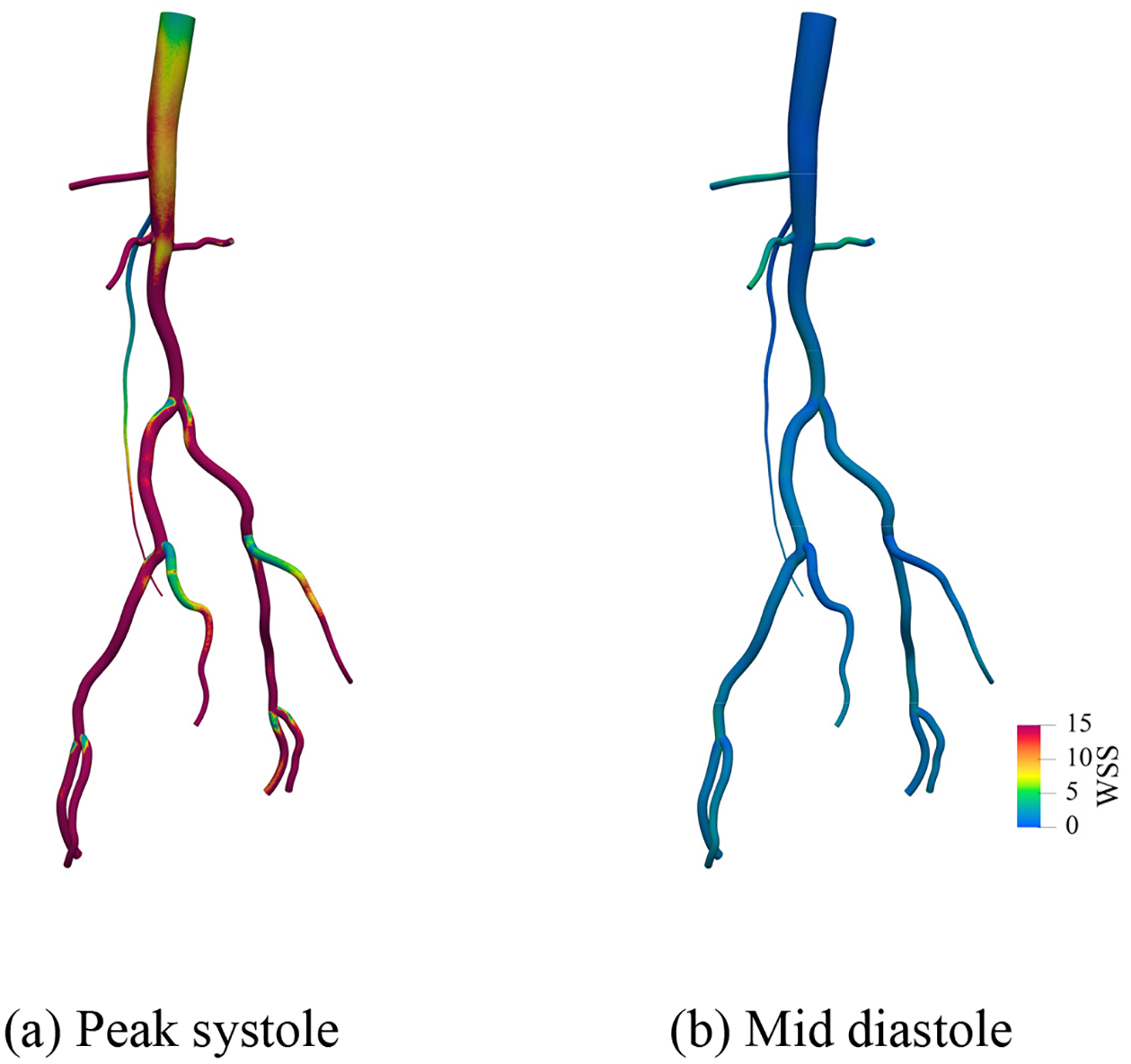
Wall shear stress (WSS) computed via the weakly imposed boundary condition at the peak systole and the mid diastole. (Unit: dyne · s/cm^2^).

Figures 25–27 present the numerical results for blood flow in the patient-specific arteries computed via the weakly imposed boundary conditions. Figure 25 shows the volume rendering of the magnitude of velocity field. Figure 26 shows the instantaneous snapshots of the viscosity field at the peak systole and the mid diastole, where the viscosity tends to be lower at the systole due to higher blood velocity. We observe the wide spread of the high-viscosity region during the diastole, especially around the thoracic aorta near the inlet, and the bifurcating parts of arteries. The high viscosity shows the propensity of blood to coagulate. Figure 27 shows the spatial distribution of the wall shear stress (WSS), which is the tangential component of the stress acting on the arterial wall, and is considered a significant factor for the progression of arterial disease. We want to note that the viscosity and WSS data is difficult to obtain via in vivo experiments. Computational methods can enable us to identify the regions where viscosity or WSS are higher, and therefore can serve as virtual platforms for patient specific care and surgical planning.

## 5. Conclusions

We have presented a stabilized method for weakly enforcing the Dirichlet boundary conditions for non-Newtonian shear-rate dependent fluids. The consistency and stabilization terms are derived by locally resolving the fine-scale variational equation along the Dirichlet boundary. For shear-rate fluids, additional boundary terms appear that are not present in the Nitsche type approaches that are otherwise employed for weak enforcement of Dirichlet boundary conditions. One of these terms is the viscosity gradient term and is a function of the shear-rate, while the other term is a zeroth-order term. The structure of the edge stabilization tensor that weights the boundary terms appears naturally, and it is free of user defined parameters. Employing edge functions the edge stabilization tensor is calculated automatically, and it adaptively adjusts itself to the magnitude of the boundary residual. Another significant advantage of the method for weakly imposed boundary conditions originates from the flexibility of not requiring nodally matched discretizations at blood-artery interaction surfaces to enforce continuity of primary fields in blood-tissue interaction problems. This relaxation in the generation of meshes for the blood and tissue sub-regions will be beneficial in complex geometries arising in patient-specific models.

The convergence rate tests show that the weakly imposed boundary conditions achieve optimal order of convergence for the velocity and pressure fields in the norms considered. Furthermore, the boundary term containing viscosity-derivative and shear-rate of the fluid is essential to obtain stable and convergent solution as a function of mesh refinement. The weakly imposed boundary conditions for 2D cavity flow outperform the strongly imposed boundary conditions in resolving the boundary layers and yield higher spatial accuracy of velocity gradients. The test case of 3D bent tube verifies the spatial accuracy of the solution in comparison with the experimental data for Newtonian fluids, and via comparison with the case of strongly imposed boundary conditions for non-Newtonian fluids. The distribution of the magnitude of the stability parameter at the boundary highlights that it adaptively adjusts itself spatially and temporally in response to the magnitude of the residual at the boundary. The test case of patient-specific arteries combines the various mathematical attributes of the method and shows its potential for the clinically relevant applications.

## Figures and Tables

**Table 1.** Edge functions to represent the fine-scale fields.

Element	Edge function
Linear triangle (T3)	4ξ(1−ξ−η)
Linear quadrilateral (Q4)	$\frac{1}{2}(\xi +1)\left(1-\eta^{2}\right)$
Quadratic triangle (T6)	$4\xi^{2}{\left(1-\xi -\eta \right)}^{2}$
Linear tetrahedron (T4)	27ξη(1−ξ−η−ζ)
Linear hexahedron (B8)	$\frac{1}{2}(1+\xi )\left(1-\eta^{2}\right)\left(1-\zeta^{2}\right)$
Quadratic tetrahedron (T10)	$27\xi^{2}\eta^{2}{\left(1-\xi -\eta -\zeta \right)}^{2}$

**Table 2.** Description of the meshes for the curved tube (h is the side length of the element).

Mesh	h	Number of nodes	Number of elements
Coarse	0.1	27,837	17,432
Fine	0.05	192,634	132,157

**Table 3.** Values of coefficients for the Carreau-Yasuda model.

$\mu_{0}$ (dyne ⋅s/cm^2^)	$\mu_{\infty }$ (dyne ⋅s/cm^2^)	λ	a	n
0.56	0.0345	1.902	1.25	0.22

**Table 4.** Range of minimum and maximum eigenvalues of the domain-interior stabilization tensor τ

Mesh	Time point	Minimum eigenvalue	Maximum eigenvalue
Coarse	$T_{A}$	$7.62\times 10^{-6}\sim 4.31\times 10^{-4}$	$8.17\times 10^{-6}\sim 5.35\times 10^{-4}$
	$T_{B}$	$9.60\times 10^{-6}\sim 5.23\times 10^{-4}$	$1.09\times 10^{-5}\sim 6.92\times 10^{-4}$
Fine	$T_{A}$	$2.73\times 10^{-6}\sim 1.97\times 10^{-4}$	$3.07\times 10^{-6}\sim 2.28\times 10^{-4}$
	$T_{B}$	$2.60\times 10^{-6}\sim 2.24\times 10^{-4}$	$3.77\times 10^{-6}\sim 2.55\times 10^{-4}$

**Table 5.** Resistance parameters (dyne ⋅s/cm^5^) for the outlets numbered in Figure 21.

Outlet	1	2	3	4	5	6, 7	8	9, 10
R	6072	420359	9676	7048	25318	6221	27848	7526

## Appendix A. Derivation of the boundary term

The domain-interior terms in the narrow band along the interface, $\tilde{\Omega }$, are converted to the boundary term on $\Gamma_{g}$ in four steps: (i) applying integration-by-parts, (ii) applying the divergence theorem and assuming the boundary term to be active only on $\Gamma_{g}$, (iii) neglecting the domain-interior term based on the assumption that $\Delta \tilde{v}\approx 0$ in $\tilde{\Omega }$, and (iv) substituting the expression for the fine-scales. Two modeling assumptions are employed in the second and third steps.

Assumption 1. The incremental fine-scales vanish, $\Delta \tilde{v}=0$, on the non-Dirichlet boundaries $\Gamma \setminus \Gamma_{g}$.

Assumption 2. The incremental fine-scales are non-zero only at the Dirichlet boundary $\Gamma_{g}$ and vanishingly small, $\Delta \tilde{v}\approx 0$, in the domain interior.

The following example shows the application of these transformations for the viscous term.

(40)
$$B=\int_{\tilde{\Omega }}2\eta \left(\dot{\gamma }\right)\left(\nabla w:\varepsilon \left(\Delta \overset{˜}{v}\right)\right)\;d\Omega$$

STEP 1: Apply the integration-by parts.

(41)
$$B=\int_{\tilde{\Omega }}\nabla \cdot \left(2\eta \left(\dot{\gamma }\right)\varepsilon \left(w\right)\cdot \Delta \overset{˜}{v}\right)\;d\Omega -\int_{\tilde{\Omega }}2\nabla \eta \left(\dot{\gamma }\right)\cdot \varepsilon \left(w\right)\cdot \Delta \overset{˜}{v}\;d\Omega -\int_{\tilde{\Omega }}2\eta \left(\dot{\gamma }\right)\left(\nabla \cdot \varepsilon \left(w\right)\right)\cdot \Delta \overset{˜}{v}\;d\Omega$$

STEP 2: Apply the divergence theorem to the first term.

(42)
$$B=\int_{\Gamma }2\eta \left(\dot{\gamma }\right)\left(\varepsilon \left(w\right)\cdot n\right)\cdot \Delta \overset{˜}{v}\;d\Gamma -\int_{\tilde{\Omega }}2\nabla \eta \left(\dot{\gamma }\right)\cdot \varepsilon \left(w\right)\cdot \Delta \overset{˜}{v}\;d\Omega -\int_{\tilde{\Omega }}2\eta \left(\dot{\gamma }\right)\left(\nabla \cdot \varepsilon \left(w\right)\right)\cdot \Delta \overset{˜}{v}\;d\Omega$$

Assuming that the incremental fine-scale field is active, $\Delta \tilde{v}\neq 0$, only at the Dirichlet boundaries $\Gamma_{g}$ (Assumption 1), the boundary term on Γ is relegated to the boundary term on $\Gamma_{g}$. Additionally, we neglect the domain-interior terms including $\Delta \tilde{v}$ based on the assumption that the fine-scale field is vanishingly small, $\Delta \tilde{v}\approx 0$, in the domain interior near the boundary $\tilde{\Omega }$ (Assumption 2).

(43)
$$B=\int_{\Gamma_{g}}2\eta \left(\dot{\gamma }\right)\left(\varepsilon \left(w\right)\cdot n\right)\cdot \Delta \overset{˜}{v}\;d\Gamma$$

STEP 3: Substituting the expression for the fine-scale in Eq (31) in the above equation, we complete the derivation of the boundary term.

(44)
$$B=-\int_{\Gamma_{g}}2\eta \left(\dot{\gamma }\right)\left(\varepsilon \left(w\right)\cdot n\right)\cdot \left(v-g\right)\;d\Gamma$$

## Appendix B. Boundedness of the boundary term involving the inverse of the shear-rate

The boundary term in Eq (33) involving the inverse of the shear-rate ${\dot{\gamma }}^{-1}$ can be written as

(45)
$$B=\int_{\Gamma_{g}}4\eta^{\prime }\left(\dot{\gamma }\right){\dot{\gamma }}^{-1}\left(\nabla w:\varepsilon \left(v\right)\right)n\cdot \varepsilon \left(v\right)\cdot \left(v-g\right)\;d\Gamma$$

In 1D case, Eq (45) can be simplified to

(46)
$$B=4\eta^{\prime }\left(\dot{\gamma }\right){\dot{\gamma }}^{-1}\frac{\partial w}{\partial x}{\left(\frac{\partial v}{\partial x}\right)}^{2}n\left(v-g\right)$$

where n=-1 or 1.

Since the shear-rate is defined by $\dot{\gamma }=\sqrt{2}|\partial v/\partial x|$,

(47)
$$B=2\sqrt{2}\eta^{\prime }\left(\dot{\gamma }\right)\frac{\partial w}{\partial x}\left|\frac{\partial v}{\partial x}\right|n\left(v-g\right)$$

We realize that this term converges to zero when the shear-rate approaches to zero, $\dot{\gamma }\to 0$. In 2D or 3D case, substituting $\dot{\gamma }=\sqrt{2}∥\varepsilon (v)∥$ in Eq (45) yields

(48)
$$B=\int_{\Gamma_{g}}2\sqrt{2}\frac{\eta^{\prime }\left(\dot{\gamma }\right)}{∥\varepsilon \left(v\right)∥}\left(\nabla w:\varepsilon \left(v\right)\right)n\cdot \varepsilon \left(v\right)\cdot \left(v-g\right)\;d\Gamma \;=\int_{\Gamma_{g}}2\sqrt{2}\eta^{\prime }\left(\dot{\gamma }\right)\left(\nabla w:\frac{\varepsilon \left(v\right)}{\sqrt{∥\varepsilon \left(v\right)∥}}\right)n\cdot \frac{\varepsilon \left(v\right)}{\sqrt{∥\varepsilon \left(v\right)∥}}\cdot \left(v-g\right)\;d\Gamma$$

where $∥\varepsilon ∥:=\sqrt{\varepsilon :\varepsilon }$. When $\dot{\gamma }\to 0,\varepsilon_{ij}\to 0$ and the term $\varepsilon (v)/\sqrt{∥\varepsilon (v)∥}$ converges to zero in the view of the L’Hopital’s rule. Therefore, the boundary term in Eq (48) also converges to zero in the limit of $\dot{\gamma }$.
